# Extracellular vesicle-packaged circBIRC6 from cancer-associated fibroblasts induce platinum resistance via SUMOylation modulation in pancreatic cancer

**DOI:** 10.1186/s13046-023-02854-3

**Published:** 2023-11-28

**Authors:** Shangyou Zheng, Qing Tian, Yuan Yuan, Shuxin Sun, Tingting Li, Renpeng Xia, Rihua He, Yuming Luo, Qing Lin, Zhiqiang Fu, Yu Zhou, Rufu Chen, Chonghui Hu

**Affiliations:** 1 Department of Pancreas Center, Department of General Surgery, Guangdong Provincial People’s Hospital (Guangdong Academy of Medical Sciences, Southern Medical University, Guangzhou, 510080 Guangdong China; 2 School of medicine, South China University of Technology, Guangzhou, 510006 Guangdong Province China; 3 Guangdong cardiovascular Institute, Guangdong Provincial People’s Hospital, Guangdong Academy of Medical Sciences, Guangzhou, 510080 Guangdong China; 4 The Second School of Clinical Medicine, Southern Medical University, Guangzhou, 510515 Guangdong China; 5 Shantou University Medical College, Shantou, 515041 Guangdong province China; 6 Guangdong Provincial Key Laboratory of Malignant Tumor Epigenetics and Gene Regulation, Sun Yat-Sen Memorial Hospital, Sun Yat-Sen University, Guangzhou, 510120 Guangdong China; 7 Department of Pancreatobiliary Surgery, Sun Yat-sen Memorial Hospital, Sun Yat-sen University, Guangzhou, 510120 Guangdong China

**Keywords:** Extracellular vesicles, Cancer-associated fibroblasts, Circular RNAs, Non-homologous end joining, SUMOylation, Oxaliplatin resistance.

## Abstract

**Background:**

Cancer-associated fibroblasts (CAFs) play pivotal roles in chemoresistance of pancreatic ductal adenocarcinoma (PDAC). However, the underlying mechanisms are poorly understood. Revealing the cross-talk network between tumor stroma and pancreatic cancer and developing effective strategies against oxaliplatin resistance are highly desired in the clinic.

**Methods:**

High-throughput sequence was used to screened the key circRNAs transmitted by extracellular vesicles (EVs) from CAFs to pancreatic cancer cells. The associations between EV-packaged circBIRC6 and chemotherapy responsiveness were validated in a cohort of 82 cases of advanced PDAC patients. Then, the effects of EV-packaged circBIRC6 on CAF-induced oxaliplatin resistance were investigated by flow cytometry, colony formation, viability of pancreatic cancer organoids in vitro and by xenograft models in vivo. RNA pulldown, RNA immunoprecipitation, and sites mutation assays were used to reveal the underlying mechanism.

**Results:**

We identified a circRNA, circBIRC6, is significantly upregulated in CAF-derived EVs and is positively associated with oxaliplatin-based chemoresistance. In vitro and in vivo functional assays showed that CAF-derived EV-packaged circBIRC6 enhance oxaliplatin resistance of pancreatic cancer cells and organoids via regulating the non-homologous end joining (NHEJ) dependent DNA repair. Mechanistically, circBIRC6 directly binds with XRCC4 and enhanced the interaction of XRCC4 with SUMO1 at the lysine 115 residue, which facilitated XRCC4 chromatin localization. XRCC4^K115R^ mutation dramatically abrogated the EV-packaged circBIRC6 induced effect. Moreover, combination of antisense oligonucleotide inhibitors against circBIRC6 with Olaparib dramatically suppressed chemoresistance in patient-derived xenograft models.

**Conclusions:**

Our study revealed that EV-packaged circBIRC6 confer oxaliplatin resistance in PDAC by mediating SUMOylation of XRCC4, introducing a promising predictive and therapeutic target for PDAC on oxaliplatin resistance.

**Supplementary Information:**

The online version contains supplementary material available at 10.1186/s13046-023-02854-3.

## Background

Pancreatic cancer, marked by high malignancy and limited treatment options, has emerged as a significant global health challenge due to its rising incidence and mortality rates [1]. Chemotherapy serves as one of the most effective means of improving prognosis for locally advanced or metastatic pancreatic cancer; for resectable or borderline resectable cases, neoadjuvant chemotherapy can benefit patients by increasing the resection rate [2]. Nonetheless, chemoresistance remains a persistent clinical obstacle in pancreatic cancer, as first-line combination therapies (gemcitabine plus capecitabine [GEMCAP] [3]. or folinic acid, 5-FU, irinotecan and oxaliplatin [FOLFIRINOX] [4]yield objective response rates of merely around 30%. Clinical evidence suggests that the most effective chemotherapy regimen for pancreatic cancer is FOLFIRINOX, with oxaliplatin being the key active agent [4]. Moreover, while targeted therapy for pancreatic cancer has shown promise, the currently proven effective agent, Olaparib, is restricted to patients exhibiting platinum sensitivity [5]. Currently, only 21% of pancreatic cancer patients are sensitive to platinum-based drugs [6]. Even for patients with germline BRCA1/2 or PALB2 mutations, the objective response rate after platinum chemotherapy is less than 50% [6]. Regrettably, the prevalence of these germline mutations in pancreatic cancer patients is less than 10% [7, 8]. Thus, investigating the mechanisms of platinum resistance in pancreatic cancer and pinpointing targets to augment platinum sensitivity may offer a breakthrough in therapy, potentially in combination with Poly ADP-ribose polymerase (PARP) inhibitors.

Chemotherapy sensitivity in pancreatic cancer patients varies greatly due to the disease’s highly heterogeneous biology and the crucial role of tumor microenvironment (TME) remodeling [9]. Studies have confirmed that the co-evolution of cancer-associated fibroblasts (CAFs) and tumor cells in the TME plays a significant regulatory role in the development of platinum resistance in pancreatic cancer [10]. CAFs can promote ovarian cancer cisplatin resistance through non-genetic mechanisms mediated by thiol metabolism regulation [11], while blocking TAK1 and TGFBR1 can significantly inhibit CAF activation, thereby enhancing the sensitivity of colon cancer cells to platinum drugs [12]. Recently, we revealed that CAFs derived from platinum-resistant patients can promote DNA damage repair by enhancing the interaction of the Ku70/Ku80 complex in pancreatic cancer cells through the secretion of IL-8, thereby inducing platinum resistance in pancreatic cancer [13]. Nonetheless, the current antagonistic treatments targeting IL-8 struggle to achieve favorable clinical outcomes, implying that alternative mechanisms could have a significant impact on CAFs-induced platinum resistance in pancreatic cancer cells.

Extracellular vesicles (EVs), ranging in diameter from 30 to 150 nm, are extracellular membranous structures that specifically enrich biomolecules such as proteins, phospholipids, DNAs, and RNAs, serving diverse biological regulatory functions within the solid TME [14]. Gaining attention as a novel means of intercellular communication, EVs can carry various bioactive molecules that modulate the activity of recipient cells. Notably, circular RNAs (circRNAs) are abundant and stable in EVs, exerting specific functions in intercellular interaction [15]. Recently, Yun et al. uncovered the vital role of exosomal circZFR, derived from CAFs, in mediating cisplatin chemoresistance in hepatocellular carcinoma by suppressing the STAT3/NF-κB pathway [16]. Nonetheless, the roles of CAF-derived EV-encapsulated circRNAs in inducing platinum resistance in pancreatic cancer remain largely unexplored, calling for further research.

In this study, we identified a specific increase in circBIRC6 (hsa_circ_0053442) levels in CAF-derived EVs, which was positively correlated with platinum resistance and poor prognosis in PDAC patients. CircBIRC6 encapsulated in CAF-derived EVs promoted XRCC4 nuclear localization via SUMOylation, consequently enhancing NHEJ repair efficiency in DNA-damaged pancreatic cancer cells, leading to platinum resistance. These findings reveal a new mechanism wherein CAFs induce platinum resistance through the transfer of EV-encapsulated, which modulates XRCC4 SUMOylation in pancreatic cancer cells, emphasizing the potential therapeutic strategy of targeting the circBIRC6/XRCC4 axis in pancreatic cancer.

## Methods

### Patient information and sample acquisition

Tissue specimens were gathered from a cohort of 82 advanced pancreatic ductal adenocarcinoma (PDAC) patients who received their primary platinum drug-based chemotherapy treatment at Guangdong Provincial People’s Hospital during 2016–2022. Prior to this initial platinum drug-based chemotherapy regimen, these patients had not undergone any other forms of chemotherapy, radiation therapy, immunotherapy, or targeted therapy. Patients specifically underwent platinum-based chemotherapy following one of the three regimens: mFOLFIRINOX, characterized by an administration of oxaliplatin, irinotecan, leucovorin on the first day and a continuous 46-hour infusion of 5-FU across the first two days, recurring every two weeks; GemOx, entailing an administration of gemcitabine on days 1 and 8, and oxaliplatin on day 1, in cycles of three weeks; or GP, which consisted of gemcitabine on days 1 and 8, and cisplatin on day 1, repeating every three weeks. The therapeutic outcomes were assessed in accordance with the Response Evaluation Criteria in Solid Tumors (RECIST), version 1.1. At the four-month mark post the commencement of chemotherapy, patients showing Complete Response (CR) or Partial Response (PR) were categorized as chemosensitive, while those with Stable Disease (SD) or Progressive Disease (PD) were labeled as chemoresistant. progression-free survival (PFS) was ascertained as the duration from the initiation of chemotherapy to the onset of disease progression.

### Extraction and cultivation of stromal fibroblasts

Both cancer-associated fibroblasts (CAFs) and primary normal adjacent tissue-associated fibroblasts (NAFs) were extracted from PDAC tissues and corresponding normal tissues. Utilizing the Human Tumor Dissociation Kit (130-095-929, Miltenyi Biotec, Germany), single cells were derived from dissociated samples. The established primary CAFs and NAFs were propagated in a fibroblast medium (FM, ScienCell) that was supplemented with 10% fetal bovine serum, 1% fibroblast growth factors, and 1% penicillin-streptomycin, and maintained at 37 °C in an atmosphere of 5% CO_2_.

### Conditioned medium preparation and co‑culture

Approximately 2 × 10^6^ stably transfected CAFs were propagated in a 10 cm cell culture dish for a duration of 48 h. The medium was subsequently retrieved, centrifuged to eliminate cell debris, and employed to culture Panc-1 and MiaPaCa-2 cells for two weeks, following which they were employed for cytological evaluations.

### Cell lines

The PDAC cell lines, Panc-1, MiaPaCa-2 and Bxpc3 procured from the American Type Culture Collection (ATCC), were maintained at 37 °C in a humidified atmosphere containing 5% CO_2_, employing Dulbecco’s Modified Eagle Medium (DMEM, Gibco) or RPMI-1640 medium (Gibco), supplemented with 10% fetal bovine serum (FBS, Gibco).

### RNA isolation and quantitative real‑time PCR (qRT‑PCR)

Total RNA was meticulously extracted from both frozen tissue samples and cultured cell lines with the aid of TRIzol reagent (Life, USA). This RNA was then converted into complementary DNA (cDNA) by employing HiScript Reverse Transcriptase (R101-01, Vazyme, China). The subsequent qRT-PCR analyses were facilitated by the utilization of the CFX96™ Real-Time System (Bio–Rad, USA) in conjunction with a TB Green Premix Ex TaqTM kit (RR820A, Takara, Japan). The relative abundance of the target gene was determined through the 2^−ΔΔCt^ method. The specific primer sequences employed in the qRT-PCR analyses are enumerated in Table S1.

### RNase R digestion and actinomycin D assay

In the context of the RNase R digestion assay, total RNA extracted from NAFs and CAFs was treated with or without 5 U/µg of RNase R (RNR07250, Epicenter Technologies), followed by an incubation period of 30 min at 37℃. For the actinomycin D assay, cells were exposed to 2 µg/mL of actinomycin D (Sigma, USA) for designated timepoints extending from 0 to 24 h. Subsequently, qRT-PCR was utilized to ascertain the expression levels of circBIRC6 and BIRC6. Each of these experiments was independently repeated thrice to ensure reliability and reproducibility.

### Cell transfection

The complete sequence of circBIRC6 was cloned into the pCD-ciR vector by IGE (Guangzhou, China). siRNA targeting SAE1and EIF4A3 and XRCC4 mutant plasmids were sourced from IGE. The shRNA constructs targeting human circBIRC6 were purchased from IGE. The siRNA and shRNA sequences are included in Table S2.

### EVs experiments

EVs were purified from CAFs culture medium as previously described. EVs was derived from the CAFs culture medium, using a pre-established procedure. Briefly, CAFs were cultivated in DMEM, supplemented with EV-free fetal bovine serum (SBI, USA) over a period of 48 h. Post-culture, the medium was collected, subjected to centrifugation at 3000 rpm at 4℃ for 10 min, aiding in the removal of cells and debris. This was succeeded by another centrifugation session at 10,000 × g for 30 min to eliminate microvesicles. After filtering the supernatant using a 0.22-µm filter, it was once again centrifuged at 120,000 × g for 70 min. The resultant pellet, rich in EVs, was rinsed with PBS and subjected to a final round of centrifugation at 120,000 × g for another 70 min. For plasma-derived EVs, we employed an EV isolation kit (Thermo Fisher Scientific, USA). We determined EV characteristics through electron microscopy utilizing negative staining and quantified them using the Nanosight LM10 (Malvern, Framingham, MA) integrated with the NTA v3.1 software (Malvern, Framingham, MA). Primary antibodies against CD9 and TSG101 were harnessed as EV markers.

For the quantification of EV-packaged RNA, we utilized an equal number of EV isolated via ultracentrifugation for RNA extraction and normalization against exogenous λ polyA (Takara, Japan) for the following qRT-PCR analysis.

### Pancreatic organoids culture

Freshly harvested human pancreatic cancer tissue was meticulously processed for organoid culture. Initial steps included sectioning the tissue and subjecting it to enzymatic digestion at a constant temperature of 37℃ for increments of 15 min, a cycle that was repeated between 3 and 5 times. The enzyme-digested tissue was then centrifuged to facilitate the collection of the supernatant. Single pancreatic cancer cells, isolated from this procedure, were thoroughly combined with matrix gel. This mixture was subsequently deposited into 24-well plates. Upon solidification of the matrix gel, a culture medium, replete with growth factors, was introduced to support organoid growth.

### Western blot

Cells were lysed using RIPA buffer, and the resultant supernatant harvested post-centrifugation. Protein concentration was quantified via a BCA protein assay kit, and equal protein volumes underwent sodium dodecyl sulfate-polyamide gel electrophoresis. Proteins were then transferred to a polyvinylidene fluoride membrane, blocked with a protein-free rapid blocking buffer, and incubated overnight with primary antibodies at 4 °C. Membranes were rinsed in TBST, treated with secondary antibodies for an hour, and immunoreactive bands were visualized with a Chemi XT4 gel imaging system. Used antibodies are cataloged in Table S3.

### CCK‑8 assay

PDAC cell drug response was evaluated by seeding 4,000 treated pancreatic cells/well in 96-well plates, followed by oxaliplatin treatment at concentrations ranging from 0 to 1000µM for 72 h. Post-incubation with 10 µl CCK-8 solution (K1018, APExBIO, USA) at 37 °C for 2 h, absorbance at 450 nm was measured using a Tecan microplate reader. Drug response was inferred from the half-maximal inhibitory concentration (IC50) values, computed via GraphPad Prism 8.0. For cell proliferation, cell viability was assessed daily via absorbance readings at 450 nm.

### Colony formation assay

700 Panc-1 or MiaPaCa-2 cells underwent designated treatments and were seeded into 6-well plates, left to attach for 24 h, and treated with fresh complete medium supplemented with 4 µM (Panc-1) or 2 µM (MiaPaCa-2) oxaliplatin. After exposed 24 h, the cells were cultured with fresh complete medium for 2 weeks. Colonies were fixed, stained, and manually counted, with three independent repetitions.

### Annexin V-PI apoptosis assay

Cell apoptosis was measured using an Annexin V-PI apoptosis detection kit (BMS500FI, Invitrogen, USA). Briefly, the cells were digested by tyrisin without EDTA and Harvested cells were resuspended in binding buffer and stained with FITC-conjugated Annexin V and PI dye for 15 min before flow cytometric analysis within an hour. This assay was performed three times.

### RNA sequencing and data analysis

Panc-1 cells at passage 5 were utilized for mRNA profiling via RNA sequencing. Post RNA extraction and cDNA library preparation, sequencing was conducted by Guangzhou Huayin Health Medical Group. Gene set enrichment analysis was executed via GSEA_Linux_4.0.3. For whole-genome transcriptome profiling post 48 h of si-circBIRC6 treatment, RNA extraction, cDNA library prep, and sequencing were carried out by BGI Technology. The clean reads were mapped onto the GRCh38.p13 reference genome using HISAT2 (v2.0.4), and a hypergeometric test-based KEGG enrichment analysis was performed via Phyper.

### Neutral comet assay

To assess DNA damage, Panc-1 and MiaPaCa-2 cells were exposed to 50 µM and 30 µM oxaliplatin, respectively, for an hour before harvesting at defined time points. A Comet Assay Kit (Trevigen, USA) was employed to conduct the neutral comet assays, following the guidelines provided by the manufacturer. The samples were subsequently stained using SYBR Gold (Invitrogen, USA) to visualize the DNA. Observation and imaging were carried out using an Olympus FluoView 500 microscope. To quantitatively assess the extent of DNA damage, the tail DNA content was evaluated using the CASP software.

### Immunofluorescence

Immunofluorescence was performed as previously described. Briefly, cells grown on confocal dishes were treated with oxaliplatin and then harvested. Post fixation and permeabilization, confocal dishes were treated with antibodies against γH2AX (ab81299, abcam, UK) overnight at 4 °C. The confocal dishes were then rinsed, treated with Alexa Fluor 488-conjugated secondary antibodies, counterstained with DAPI, and visualized via confocal fluorescence microscopy (Carl Zeiss AG, Germany).

### pimEJ5-GFP reporter assay

The pimEJ5-GFP reporter assay was executed as previously delineated. Tumor cells underwent transfection with pimEJ5-GFP plasmid using Lipofectamine 3000. Following this, puromycin was utilized to select and establish stable clones. Cells with stable pimEJ5-GFP expression were subsequently transfected with pCBASceI (I-SceI) plasmids using Lipofectamine 3000. After a period of 48–72 h post-transfection, GFP-positive cells were analyzed by Fluorescence Activated Cell Sorting (FACS). The pimEJ5-GFP plasmid was generously provided by Dr. Jeremy Stark (Addgene plasmid #44,026).

### Fluorescence in situ hybridization (FISH)

The spatial distribution of circBIRC6 in Cancer-Associated Fibroblasts (CAF) was probed using a FISH Kit (Gene Pharma, Suzhou, China). Cells were fixed, and following the manufacturer’s protocol, were subjected to overnight hybridization with Cy3-labeled circBIRC6 probes at 37 °C. Nuclei staining was carried out with DAPI. Fluorescent signals were captured by confocal fluorescence microscopy (Carl Zeiss AG, Germany). Probe sequences can be found in Table S2.

### Subcellular fractionation

Cytoplasmic and nuclear RNA fractions were obtained using a PARIS Kit (Thermo Scientific, USA), according to the provided protocol. Subsequently, the nuclear to cytoplasmic RNA ratio was determined by quantitative Reverse Transcription Polymerase Chain Reaction (qRT-PCR), with U6 and GAPDH serving as nuclear and cytoplasmic controls, respectively. Primer sequences are available in Table S1. For protein fractions, a subcellular protein fractionation kit (Thermo Scientific, USA) was used following the manufacturer’s protocol, and relative expression of protein in the cytoplasm and nucleus was determined by western blot.

### RNA pull-down assays

Biotin-labeled circBIRC6 was purchased from IGE. RNA pull-down assays were conducted using a Magnetic RNA-Protein Pull-Down Kit (Thermo Scientific, USA). In brief, biotinylated circBIRC6, immobilized on streptavidin magnetic beads, was incubated with nuclear extracts at 4 °C overnight. Subsequently, the protein bound to circBIRC6 was eluted and analyzed either by mass spectrometry or western blot. Silver staining was executed using a Pierce Silver Stain Kit (Thermo Scientific, USA), according to the manufacturer’s instructions.

### Co-immunoprecipitation (Co-IP)

Co-IP was undertaken using a Pierce Co-IP Kit (Thermo Scientific, USA), adhering to the manufacturer’s guidelines. Briefly, harvested cells were lysed in IP buffer. A solution of 10 µg anti-SAE1/anti-XRCC4 or control IgG was combined with the resin and incubated with a 500 µg protein mixture at 4 °C overnight, while being gently rotated. Following three wash cycles, proteins were extracted for subsequent western blot analysis.

### Immunohistochemistry

Immunohistochemistry (IHC) was conducted using paraffin-embedded specimens as previously documented. Briefly, post-deparaffinization, rehydration, and heat-induced antigen retrieval, specimens were incubated with γ-H2AX-specific antibodies overnight at 4 °C. Specimens were then washed and incubated with secondary antibodies, followed by exposure to DAB developer and hematoxylin.

### DAB Tunel Cell apoptosis detection

DAB tunel cell apoptosis detection was carried out using a DAB Tunel Cell Apoptosis Detection kit (Servicebio, Wuhan), following the manufacturer’s instructions. Briefly, after deparaffinization, rehydration, Proteinase K digestion, and blocking of endogenous peroxides, specimens were incubated with Equilibration Buffer for 10 min. Specimens were then labeled with TdT incubation buffer (Recombinant TdT enzyme: Biotin-dUTP Labeling Mix: Equilibration Buffer = 1:5:50) for 1 h at 37℃, and subsequently stained with DAB developer and hematoxylin following Streptavidin-HRP reaction.

### In situ hybridization (ISH)

ISH was conducted using a double-DIG labeled circBIRC6 probe (IGE, Guangzhou, China) to detect the expression of circBIRC6. Following deparaffinization, rehydration, and proteinase K digestion, the samples were subjected to overnight probe hybridization at 37℃. The hybridization signal was subsequently identified with an Enhanced Sensitive ISH Detection Kit (MK1032, BOSTER, China), as per the manufacturer’s guidelines. Probe sequences are listed in Table S2.

### In vivo study

Orthotopic xenograft tumor models were established by introducing 1*10^5 luc-Panc-1 cells per group, which were subsequently treated following the protocols used for subcutaneous models. After 12 days post-implantation, therapeutic interventions were initiated, at which point the first set of in vivo imaging system (IVIS) images was captured. A second round of IVIS imaging was conducted following a 15-day treatment course, after which the tumors were excised. At each point of the IVIS assessment, intraperitoneal injections of D-Luciferin, potassium Salt (150 mg/kg, 40902ES01, Yeasen, China) were administered, facilitating the capture of orthotopic fluorescence images with an in vivo FX PRO system (BRUKER Corporation, USA). Notably, throughout the course of the in vivo study, investigators were blinded to the group assignments during the evaluation of experimental outcomes.

Primary tumor samples procured from oxaliplatin-resistant PDAC patients undergoing surgical procedures were utilized to establish subcutaneous tumors in 4-week-old NOD/SCID/gamma (NSG) mice (F1 generation). Xenografts harvested from these F1 mice were segmented into small sections and subsequently implanted into a second generation of mice (F2). Once these F2 tumors reached a volume of approximately 1500 mm^3^, they were surgically removed, segmented, and transplanted into a third generation of mice (F3). When the volume of the xenografts reached around 200 mm^3^, the subjects received a combination treatment regimen of in vivo antisense oligonucleotide (ASO) inhibitors against circBIRC6, the PARP1 inhibitor Olaparib, and oxaliplatin-based chemotherapy. For the delivery of these treatments, in vivo-optimized oxaliplatin (10 mg/kg, intraperitoneal injection), ASO targeting circBIRC6 (5 mg/kg, intravenous injection), or Olaparib (50 mg/kg, intraperitoneal injection) were used as per the experiment’s requirements. Tumor volumes were tracked on a weekly basis and subsequently subjected to further IHC analyses. The evaluation of chemotherapy responses was performed according to human clinical assessment standards.

All animal experiments were executed in strict adherence to the guidelines ratified by the Animal Experimental Research Ethics Committee of South China University of Technology.

### Bioinformatic analysis

The secondary structure of circBIRC6 was inferred using the RNAalifold tool (http://rna.tbi.univie.ac.at/cgi-bin/RNAWebSuite/RNAalifold.cgi). The binding motifs for XRCC4 were obtained from the POSTAR2 database (http://lulab.life.tsinghua.edu.cn/postar2). The potential SUMO2 binding site on XRCC4 was predicted using the GPS-SUMO bioinformatics tool. Schematic illustration was created with BioRender.com.

### Statistical analysis

The animal study incorporated five biological replicates, whereas the remaining experiments employed three biological replicates. Sample sizes were established to provide sufficient statistical power to detect a predefined effect size, predicated on outcomes of initial investigations and pilot experiments. All statistical computations were carried out using SPSS 12.0 software (IBM Corp, USA) and GraphPad Prism version 8.0 (GraphPad Software, USA). Differences between two groups were determined using Student’ s t-test, while one-way analysis of variance (ANOVA) was utilized for comparisons among multiple groups. Survival curves were evaluated using the Kaplan-Meier method complemented by a log-rank test. All error bars denote the mean ± standard deviation (SD). A p-value < 0.05 was considered indicative of statistical significance.

## Results

### circBIRC6 is associated with a poor oxaliplatin response in PDAC

**Fig. 1:**
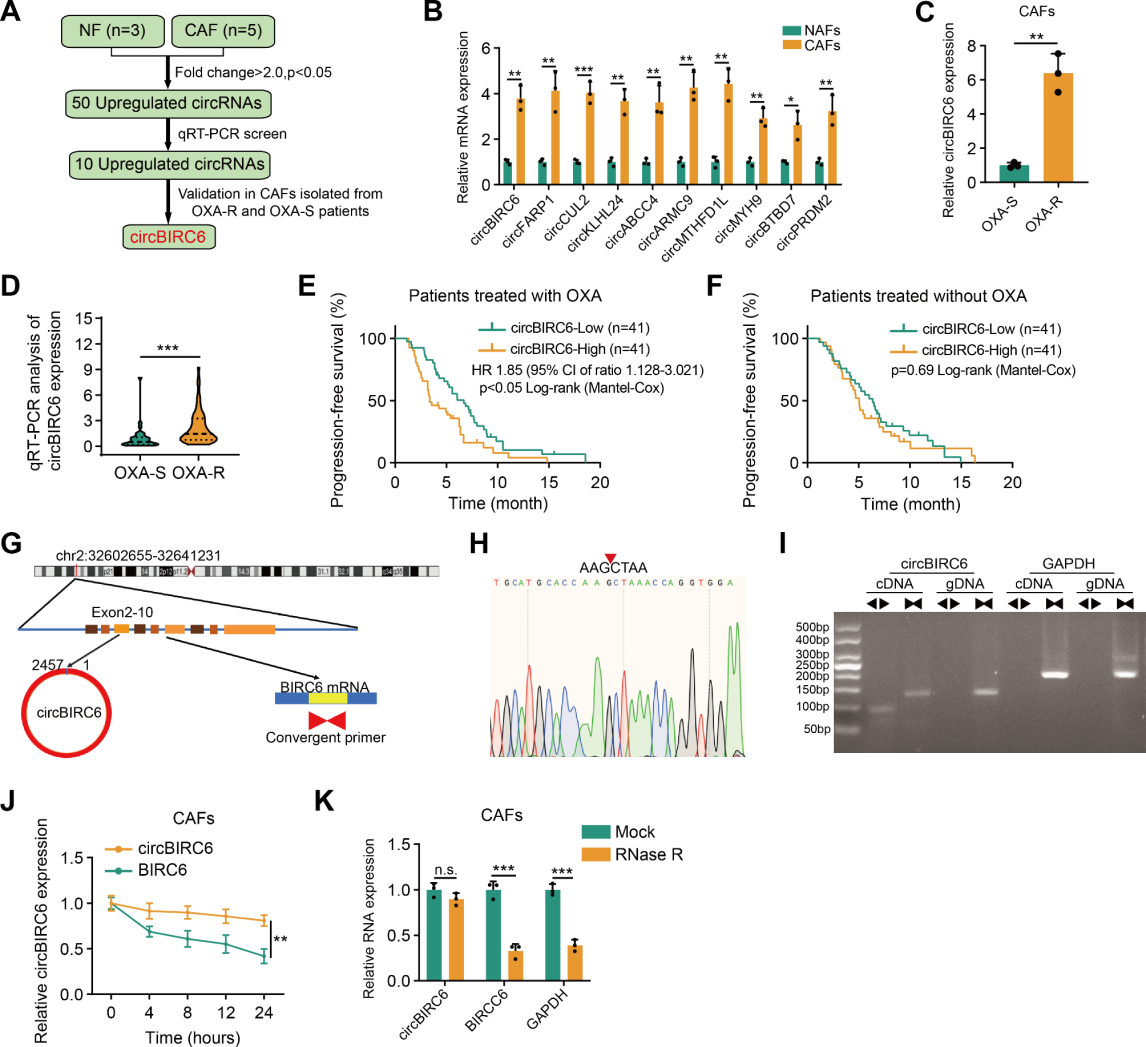
Correlation of circBIRC6 with response to oxaliplatin-based chemotherapy. **(A)** Graphical representation of the process used to identify elevated levels of circBIRC6 in CAFs from oxaliplatin-resistant patients. **(B)** qRT-PCR analysis of 10 selected circRNAs in both NAFs and CAFs (n = 3). **(C)** Comparative analysis of circBIRC6 expression in CAFs obtained from oxaliplatin-resistant (OXA-R) and -sensitive (OXA-S) pancreatic cancer patients via qRT–PCR (n = 3). **(D)** qRT-PCR evaluation of circBIRC6 expression in OXA-S (n = 31) and OXA-R (n = 51) PDAC samples, depicted as violin plots. **(E-F)** Kaplan–Meier survival analysis for advanced PDAC patients receiving oxaliplatin-based (E) or non-platinum chemotherapy (F), stratified by high or low circBIRC6 expression. **(G)** Genomic loci diagram depicting the exonic origin (exons 2 to 10) of circBIRC6. **(H)** Validation of the back-splice junction of circBIRC6 using Sanger sequencing. **(I)** Amplification of cDNA and gDNA in CAFs using convergent and divergent primers, with GAPDH as a negative control. **(J)** Temporal analysis of circBIRC6 and BIRC6 mRNA expression in CAFS post-treatment with actinomycin D. **(K)** Comparative PCR analysis of circBIRC6, BIRC6, and GAPDH expression in CAFs following RNase R treatment. Data are mean ± SD. n.s., not significant. *p < 0.05, **p < 0.01, ***p < 0.001

To investigate the key circRNAs involved in the cross-talk between cancer-associated fibroblasts (CAFs) and tumor cells in platinum-based chemotherapy resistance, we conducted high-throughput sequencing (GSE172096) on CAFs and their paired normal adjacent tissue-associated fibroblasts (NAFs) (Fig. 1A). qRT-PCR analysis verified that 10 out of 50 upregulated circRNAs (fold-change > 2 and P < 0.05) showed a significant increase in CAFs (Fig. 1B). Among these circRNAs, circBIRC6 exhibited significantly higher expression in CAFs and tumor tissues of oxaliplatin-resistant patients than in those of oxaliplatin-responsive patients (Fig. 1C-D). Furthermore, high expression of circBIRC6 was significantly correlated with shorter PFS in the pancreatic cancer patients who received platinum-based chemotherapy (Fig. 1E). Interestingly, there was no significant difference in PFS regardless of the circBIRC6 level in the patients who received other chemotherapeutic regimens without platinum (Fig. 1F). To elucidate whether circBIRC6 originates from CAFs or tumor cells and contributes to an unfavorable prognosis, we isolated CAFs and their paired tumor cells from pancreatic cancer patients undergoing platinum-based chemotherapy. circBIRC6 expression was notably elevated in CAFs compared to tumor cells (Figure S1A). Crucially, with increasing passages, circBIRC6 expression in tumor cells diminished, implying its primary origin in CAFs (Figure S1B). Additionally, circBIRC6 expression in tissues showed a positive correlation with circBIRC6 levels in CAFs (Figure S1C). In summary, our data suggest that CAF-derived circBIRC6 is the predominant source in tissues, contributing to reduced PFS in pancreatic cancer patients treated with platinum-based chemotherapy.

To determine the origin of circBIRC6, we consulted the UCSC Genome Browser and discovered that circBIRC6 originated from exon 2 to 10 of the BIRC6 gene (chr2: 32,602,655–32,641,231) (Fig. 1G). Employing Sanger sequencing and nucleic acid electrophoresis assays, we confirmed the existence of circBIRC6, which could be amplified solely from cDNA and not gDNA (Fig. 1H-I). Moreover, circBIRC6 demonstrated increased resistance to RNase-R digestion and a longer half-life compared to its parental linear transcript in CAFs upon treatment with RNase R and actinomycin D (Fig. 1J-K). These findings indicate that circBIRC6 possesses a covalently closed continuous loop structure and is associated with oxaliplatin resistance.

### EIF4A3 promotes the biogenesis of circBIRC6

We further sought to elucidate the molecular mechanisms driving circBIRC6 up-regulation in platinum-resistant PDAC. Interactions between RNA-binding proteins (RBPs) and circRNAs regulate circRNA generation [17, 18]. We predicted potential RBPs that bind to circBIRC6 using CircInteractome (Figure S2A). Among these candidates, EIF4A3 expression was significantly higher in CAFs from platinum-resistant pancreatic patients than in those from sensitive ones (Figure S2B). Depletion of EIF4A3 markedly reduced circBIRC6 expression in CAFs from oxaliplatin-resistant patients, whereas its overexpression had the inverse effect in CAFs from oxaliplatin-sensitive patients (Figure S2C-D). Furthermore, RIP assay confirmed the binding of the EIF4A3 protein to circBIRC6 (Figure S2E). These findings suggest that EIF4A3 plays a pivotal role in promoting circBIRC6 synthesis.

### circBIRC6 was packaged into EVs and transmitted to Tumor cells

**Fig. 2:**
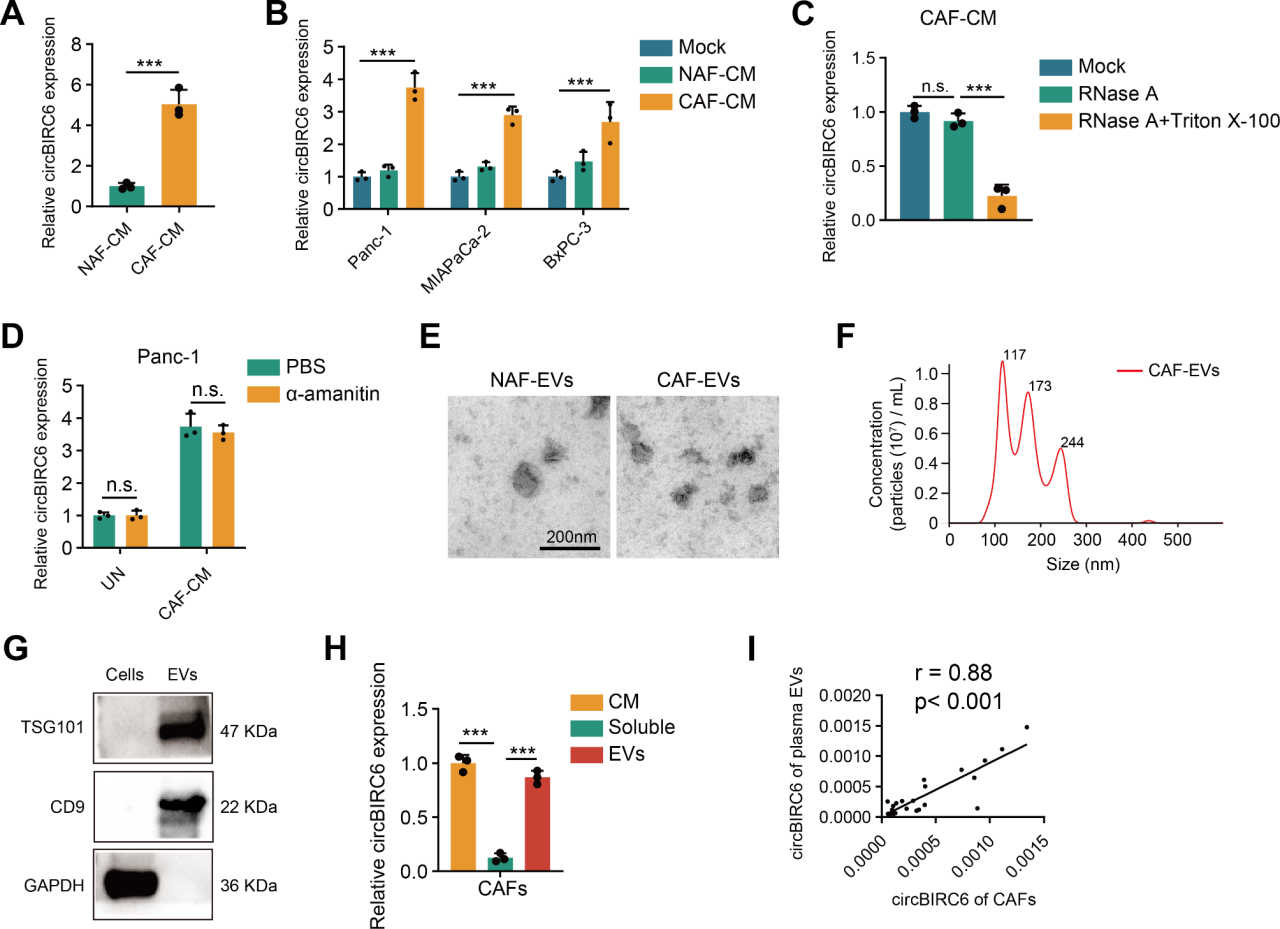
CAF-secreted EVs exhibit elevated levels of circBIRC6. **(A)** Assessment of circBIRC6 expression in the culture medium of CAFs and NAFs via qRT-PCR. **(B)** Quantification of circBIRC6 expression in Panc-1, MiaPaCa-2, and BxPC-3 cells post-treatment with culture medium from CAFs and NAFs. **(C)** Analysis of circBIRC6 levels in the CAF culture medium following treatment with RNase or a combination of RNase and Triton X-100. **(D)** qRT-PCR analysis of circBIRC6 expression in Panc-1 and MiaPaCa-2 cells following treatment with CAF culture medium in the presence of α–amanitin, an RNA transcription inhibitor. **(E-F)** Characterization of CAF-derived EVs using TEM (E) and NanoSight (F). Scale bars represent 200 nm. **(G)** Western blot detection of EV-derived protein markers (TSG101 and CD9) in CAF cell lysates and EVs. **(H)** Comparative analysis of circBIRC6 levels in EVs, soluble fractions, and whole culture medium from CAFs using qRT-PCR (n = 3). **(I)** Correlation analysis between EV-packaged circBIRC6 expression in CAFs and patient plasma samples. Data are mean ± SD. n.s., not significant. ***p < 0.001

Interestingly, we found that circBIRC6 levels were higher in the culture medium (CM) of CAFs compared to NAFs (Fig. 2A). A remarkable increase in circBIRC6 levels was detected in PDAC cells co-cultured with CAFs relative to those co-cultured with NAFs or cultured alone (Fig. 2B). Subsequently, we further investigated the extracellular circBIRC6 pattern. RNase treatment did not impact circBIRC6 levels in CAF-CM, while combined RNase and Triton X-100 treatment significantly reduced circBIRC6 levels in CAF-CM (Fig. 2C). Moreover, treating Panc-1 cells with α-amanitin to inhibit endogenous transcriptional activation did not affect the elevation of circBIRC6 induced by CAF-CM (Fig. 2D), indicating that extracellular circBIRC6 could be transmitted to tumor cells.

To confirm that extracellular circBIRC6 was mainly membrane-enclosed, we isolated EVs from CAF-CM. Transmission electron microscopy, NanoSight analysis and Western blot verified the typical morphology, size, and biomarkers of EVs (Fig. 2E-G). The level of circBIRC6 in EVs was almost equivalent to that in CAF-CM (Fig. 2H), implying that circBIRC6 was enriched in CAF-secreted EVs rather than being directly released. Furthermore, plasma EV-packaged circBIRC6 levels exhibited a positive association with EV-packaged circBIRC6 derived from paired primary CAFs (Fig. 2I), suggesting that plasma EV-packaged circBIRC6 primarily originated from CAFs in the PDAC TME.

### CAF-derived EV-packaged circBIRC6 promotes oxaliplatin resistance in vitro

**Fig. 3:**
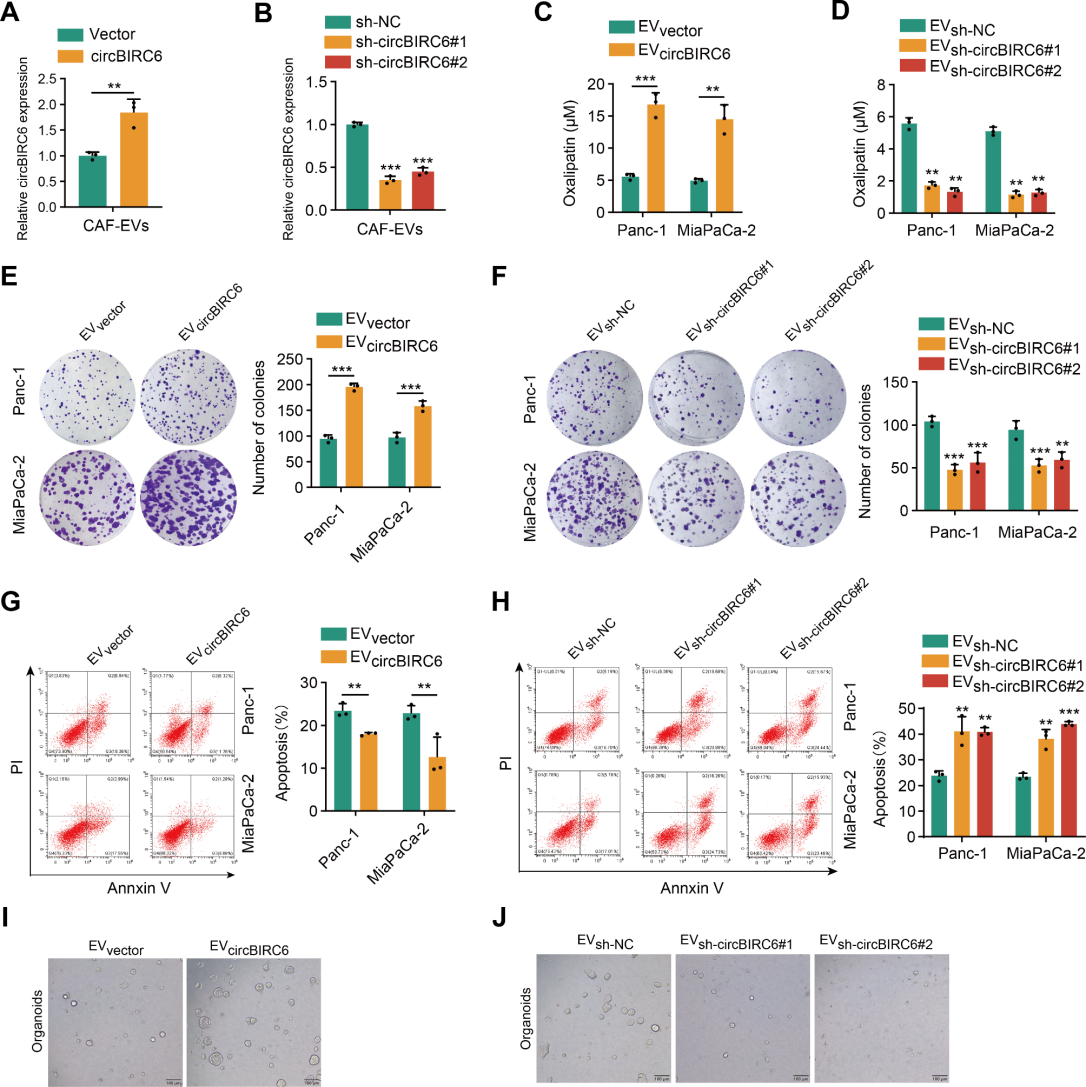
circBIRC6 transmitted by EVs enhanced oxaliplatin resistance in pancreatic cancer cells. **(A-B)** qRT-PCR analysis demonstrating levels of EV-packaged circBIRC6 in CAFs overexpressing **(A)** or depleted of **(B)** circBIRC6. **(C-H)** Panc-1 and MiaPaCa-2 cells exposed to CAF-derived EVs for a week, post which experimental analyses were conducted. (C-D) IC50 value of oxaliplatin-treated pancreatic cancer cells assessed using CCK-8. **(E-F)** Imaging and quantification of colonies formed by the treated pancreatic cancer cells. **(G-H)** Imaging and quantification of apoptosis in treated pancreatic cancer cells through flow cytometry. **(I-J)** Representative organoids derived from pancreatic cancer patients under the specified treatments. Data are expressed as mean ± SD. **p < 0.01, ***p < 0.001

To explore the role of CAF-derived EV-packaged circBIRC6 in modulating oxaliplatin resistance in vitro, we isolated EVs from CAFs transfected with a circBIRC6 expression plasmid or lenti-circBIRC6-shRNA. Silencing circBIRC6 substantially diminished the circBIRC6 levels in the EVs secreted by CAFs, while overexpressing circBIRC6 produced the opposite effect (Fig. 3A-B). Subsequently, we next incubated PDAC cells were with these EVs for 6 days. CCK-8 and colony formation assays revealed that PDAC cells incubated with EVs derived from circBIRC6-overexpressing CAFs displayed elevated IC50 values and enhanced survival rates compared to the control group upon oxaliplatin treatment, whereas silencing circBIRC6 abrogated these effects (Fig. 3C-F and Figure S3A-D). Additionally, treatment with EVs derived from circBIRC6-overexpressing CAFs attenuated oxaliplatin-induced apoptosis in PDAC cells, while silencing circBIRC6 had opposite effects (Fig. 3G-H). Moreover, Pancreatic cancer organoids treated with EVs from circBIRC6-overexpressing CAFs exhibited enhanced proliferation (Fig. 3I). Conversely, circBIRC6 silencing led to reduced proliferation (Fig. 3J). We utilized previously established platinum-resistance pancreatic cancer cell lines to explore the effect of circBIRC6 on established platinum resistance. IC50 assays showed that EVs from CAFs elevated the IC50 values in platinum-resistant tumor cells. Knockdown of circBIRC6 mitigated this effect, yet it did not overcome the inherent platinum resistance (Figure S3D-E). Collectively, these findings suggest that EV-packaged circBIRC6 plays a pivotal role in conferring oxaliplatin resistance in vitro.

### NHEJ pathway is responsible for circBIRC6-mediated oxaliplatin resistance

**Fig. 4:**
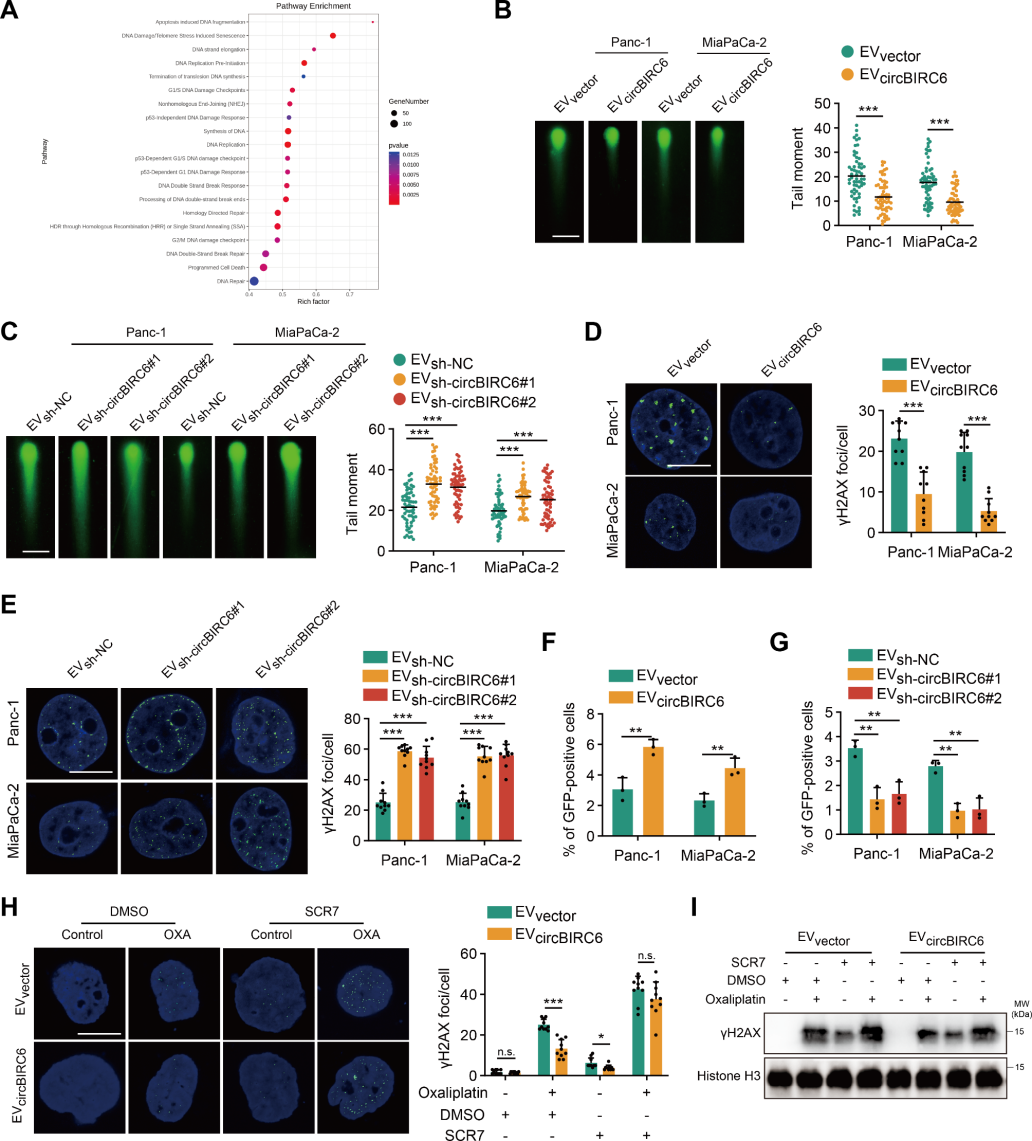
EV-packaged circBIRC6 promotes oxaliplatin resistance via activation of NHEJ-dependent DNA repair in pancreatic cancer. **(A)** Pathway enrichment analysis of differentially expressed mRNAs in Panc-1 cells treated with EVs from circBIRC6-overexpressing CAFs. **(B-G)** Panc-1 and MiaPaCa-2 cells treated with CAF-derived EVs for a week were analyzed as follows: **(B-C)** Imaging and quantification of oxaliplatin-induced DNA damage using a neutral comet assay. Scale bar, 200 μm. **(D-E)** Imaging and quantification of γH2AX-positive foci. Scale bar, 10 μm. **(F-G)** NHEJ-mediated DNA repair efficiency evaluated via pDR-GFP reporter assay. **(H-I)** Panc-1 and MiaPaCa-2 cells, post-treatment with EVs from circBIRC6-overexpressing CAFs and exposed to oxaliplatin, were allowed to recover with or without 10 µM SCR7. **(H)** Imaging and quantification of DNA damage using a neutral comet assay. Scale bar, 10 μm. **(I)** Western blot analysis of γH2AX expression. Data are expressed as mean ± SD. *p < 0.05, **p < 0.01, ***p < 0.001

To further elucidate the mechanism underlying EV-packaged circBIRC6-driven oxaliplatin resistance, we performed transcriptome sequencing in PANC-1 cells treated with EVs derived from either circBIRC6-depleted CAFs or control. Kyoto Encyclopedia of Genes and Genomes (KEGG) analysis revealed enrichment of the DNA repair pathway in Panc-1 cells treated with EVs derived from shNC-CAF compared to circBIRC6-depleted CAFs (Fig. 4A). Double-strands DNA break (DSB) repair is known to be one of the critical mechanisms for oxaliplatin resistance. We subsequently investigated whether EV-packaged circBIRC6 altered the efficiency of DSB repair in PDAC cells using neutral comet assays. Shorter comet tails were observed in PDAC cells treated with EVs derived from circBIRC6-overexpressing CAFs (Fig. 4B). Conversely, silencing circBIRC6 attenuated the effect of CAF-derived EVs on reducing comet tail lengths (Fig. 4C). Consistently, the levels of γH2AX in PDAC cells treated with EVs derived from circBIRC6-overexpressing CAFs were lower than in the control group, while silencing circBIRC6 had the opposite effect (Fig. 4D-E). HR and NHEJ are key pathways for DSB repair. We found that circBIRC6 enhanced the efficiency of NHEJ but not HR (Fig. 4F-G and Figure S4A-B). We then investigated how the NHEJ pathway, inhibited by SCR7, played a crucial role in circBIRC6-mediated oxaliplatin resistance. The CCK-8 cytotoxicity and colony formation assays showed that SCR7 negated the proliferative advantage conferred by EV-packaged circBIRC6 to tumor cells (Figure S4C-D). Moreover, SCR7 counteracted the anti-apoptotic effect induced by EV-packaged circBIRC6 (Figure S4E). γH2AX analysis revealed that blocking the NHEJ pathways significantly weakened circBIRC6’s positive impact on DNA repair efficiency (Fig. 4H-I). Our findings suggest that circBIRC6 mediates oxaliplatin resistance through the NHEJ pathway, and blocking NHEJ pathway abolished the effect of circBIRC6 on oxaliplatin resistance.

### circBIRC6 directly binds to XRCC4

**Fig. 5:**
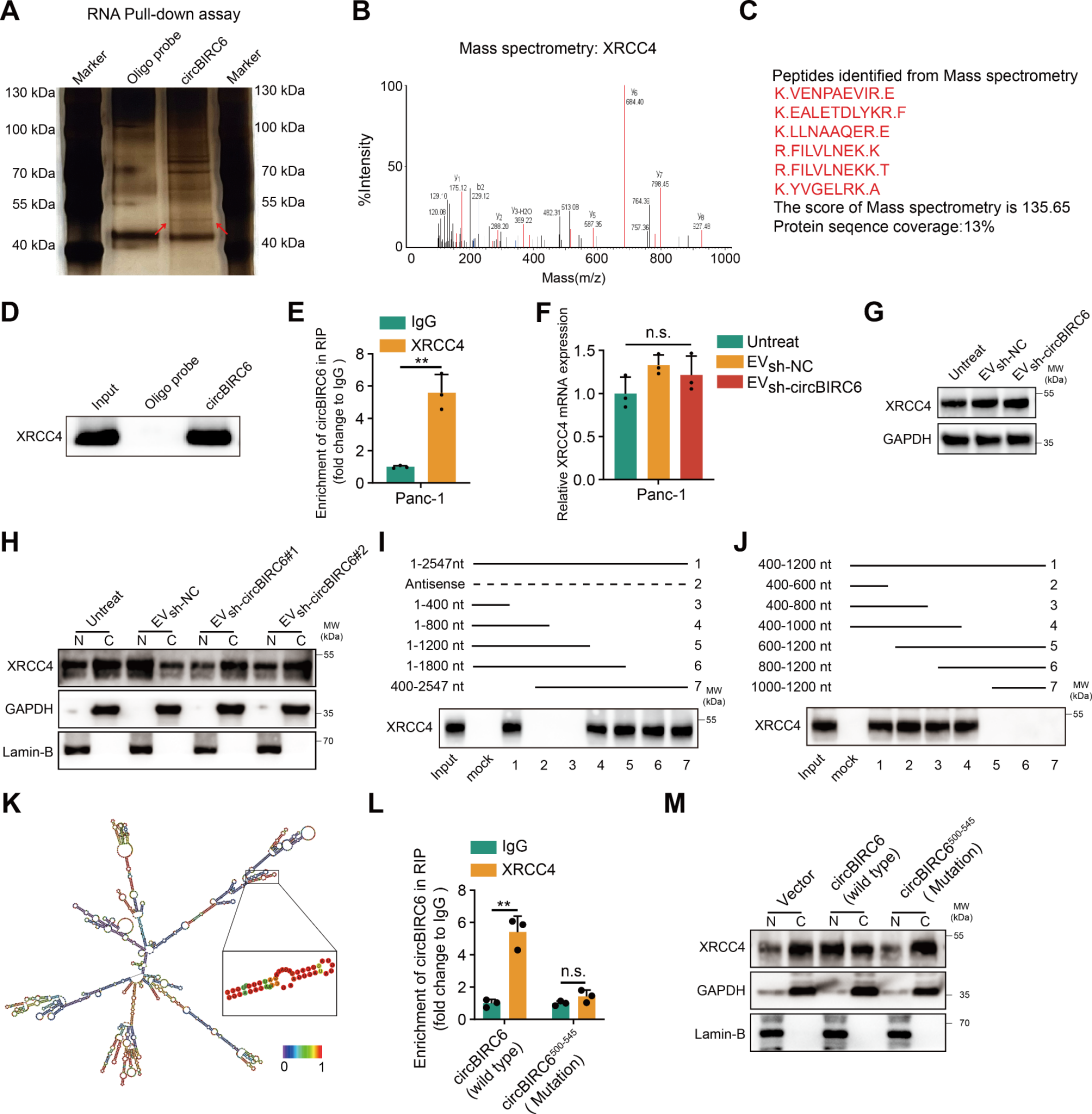
circBIRC6 direct interaction with XRCC4 enhances its chromatin localization. **(A)** Silver staining of RNA pull-down assay revealing the distinct band associated with the biotin-labeled circBIRC6 probe in Panc-1 cells treated with CAF-derived EVs. **(B-D)** Mass spectrometry (B-C) and western blot (D) analysis identify XRCC4 as a potential circBIRC6 interacting protein. **(E)** RIP assay validating circBIRC6’s interaction with XRCC4 in Panc-1 cells treated with CAF-derived EVs. **(F-G)** mRNA and protein levels of XRCC4 in Panc-1 cells post-treatment with EVs from CAFs transfected with lenti-circBIRC6-shRNA or lenti-NC-shRNA. n.s., not significant. **(H)** Western blot examination of the cytoplasmic and nuclear distribution of XRCC4 in Panc-1 cells treated with EVs from CAFs transfected with lenti-circBIRC6-shRNA or lenti-NC-shRNA. **(I-J)** In vitro RNA/protein interaction assay between biotin-labeled circBIRC6 fragments and recombinant XRCC4 protein. **(K)** RNAalifold-based prediction of circBIRC6 secondary structure. The prediction was based on minimum free energy (MFE). Color scale indicates prediction confidence. Red shades reflect strong prediction confidence. **(L)** The binding of wild-type or mutated circBIRC6 to XRCC4 was evaluated by RNA immunoprecipitation (RIP) in Panc-1 cells. n.s., not significant. **(M)** The subcellular distribution of XRCC4 in the cytoplasm and nucleus of Panc-1 cells transfected with wild-type or mutated circBIRC6 was examined using Western blot analysis. Data are expressed as mean ± SD. **p < 0.01

To determine the mechanism by which circBIRC6 enhanced the efficiency of NHEJ repair, we performed RNA pulldown assays to identify potential binding proteins in Panc-1 cells. A distinct band was observed by silver staining at approximately 55 − 40 kDa, enriched by the biotinylated circBIRC6 probes (Fig. 5A). This distinct band was validated as XRCC4 via mass spectrometry and Western blotting (Fig. 5B-D). The interaction between circBIRC6 and XRCC4 was further substantiated through an RNA immunoprecipitation (RIP) assay (Fig. 5E). Silencing circBIRC6 in CAFs did not impact XRCC4 mRNA and protein levels in Panc-1 cells treated with CAF-derived EVs (Fig. 5F-G). Surprisingly, we observed that CAF-derived EVs enhanced XRCC4 recruitment to chromatin, which was suppressed following circBIRC6 depletion (Fig. 5H). Next, we generated a series of sequential deletions of circBIRC6 and determined that the 400–600 nt region of circBIRC6 was crucial for XRCC4 interaction (Fig. 5I-J). Noncoding RNAs typically interact with proteins via stem-loop structures. We predicted that the 500–545 nt region of circBIRC6 (circBIRC6^500–545^) forms a double-stranded stem-loop structure (Fig. 5K). Deletion mutation of the 500–545 nt region of circBIRC6 impaired the enhanced circBIRC6 enrichment in the XRCC4 RIP assay and XRCC4 chromatin localization (Fig. 5L-M).

### EV-packaged circBIRC6 regulates XRCC4 chromatin localization via SUMOylation

**Fig. 6:**
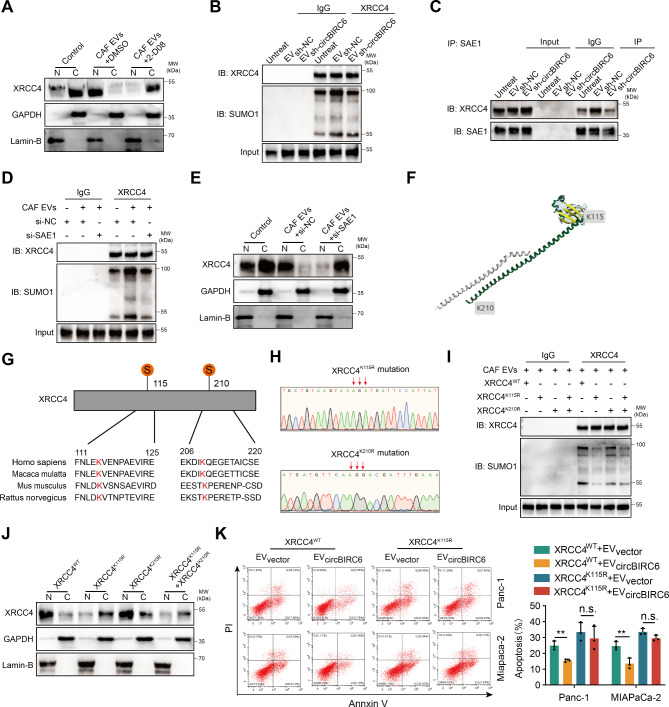
circBIRC6 augments SUMOylation of XRCC4 at K115 residue. **(A)** Western blot analysis illustrating cytoplasmic and nuclear distribution of XRCC4 in Panc-1 cells treated with EVs isolated from CAFs and the SUMOylation inhibitor (2-D08). **(B)** Co-immunoprecipitation (Co-IP) study presenting the SUMO1 binding on XRCC4 in Panc-1 cells treated with EVs from CAFs transfected with lenti-circBIRC6-shRNA or lenti-NC-shRNA. **(C)** Co-IP assays elucidating the interaction between XRCC4 and SAE1 in Panc-1 cells treated with EVs from CAFs transduced with lenti-circBIRC6-shRNA or lenti-NC-shRNA. **(D)** Co-IP analysis of SUMO1 binding on XRCC4 in Panc-1 cells transfected with SAE1 siRNA and treated with EVs from CAFs. **(E)** Western blot analysis of cytoplasmic and nuclear distribution of XRCC4 in Panc-1 cells transfected with SAE1 siRNA and treated with EVs from CAFs. **(F)** Schematic depiction of the predicted SUMO1 binding sites on XRCC4 obtained via GST-SUMO. **(G)** Sequence alignment of XRCC4 homologs across various species. **(H)** Sequencing evaluation of the XRCC4^K115R^ and XRCC4^K210R^ mutations. **(I)** Co-IP assays assessing the SUMO1 binding sites on XRCC4. **(J)** Western blot analysis of cytoplasmic and nuclear distribution of XRCC4 in Panc-1 cells transfected with XRCC4 ^K115R^ or XRCC4^K210R^ mutations and treated with EVs from CAFs. **(K)** Representative image and quantification of apoptosis in Panc-1 cells transfected with XRCC4^K115R^ mutations and treated with EVs from CAFs using flow cytometry. n.s., not significant. Data are expressed as mean ± SD. **p < 0.01

Our pervious study, along with others, has demonstrated the significance of post-translational modifications (PTMs) in protein localization. To identify the crucial PTM involved in XRCC4 chromatin enrichment, Panc-1 cells were treated with CAF-derived EVs and various PTM inhibitors. Inhibition of SUMOylation, but not other types of PTMs, significantly abrogated the chromatin localization of XRCC4 (Fig. 6A and Figure S5A). Moreover, Co-IP assays further corroborated that silencing circBIRC6 markedly reduced the attachment of SUMO1, a SUMOylation modifier, to XRCC4 (Fig. 6B). Additionally, silencing circBIRC6 did not significantly alter the ubiquitination, phosphorylation, or methylation of XRCC4 (Figure S5B-D). This evidence supports the notion that circBIRC6 primarily affects XRCC4’s SUMOylation and not its other PTM. Previous research reported that SAE1, an E1 SUMO-activating enzyme, is crucial for SUMOylation modification. Co-IP assays demonstrated that circBIRC6 strengthened the interaction between XRCC4 and SAE1 (Fig. 6C). Furthermore, silencing SAE1 significantly mitigated SUMO1 modification of XRCC4 and inhibited XRCC4 chromatin localization (Fig. 6D-E). Given the vital role of modification residues in determining SUMOylation’s biological impact on target proteins, we utilized GPS-SUMO, a tool for SUMOylation site prediction, identifying two potential SUMO modification sites on XRCC4 (K115 and K210) (Fig. 6F-G). The K115R mutation but not K210R mutation dramatically obstructed XRCC4 SUMOylation and chromatin enrichment (Fig. 6H-J). We then investigated whether SUMOylation-dependent XRCC4 chromatin localization is necessary for EV-packaged circBIRC6-mediated oxaliplatin resistance. The K115R mutation of XRCC4 counteracted the EV-packaged circBIRC6-induced oxaliplatin resistance in Panc-1 and MiaPaCa-2 cells (Fig. 6K). Taken together, these findings suggest that EV-packaged circBIRC6 promotes oxaliplatin resistance by facilitating the recruitment of XRCC4 to chromatin through SUMOylation modification.

### EV-packaged circBIRC6 induces oxaliplatin resistance in pancreatic cancer in vivo

**Fig. 7:**
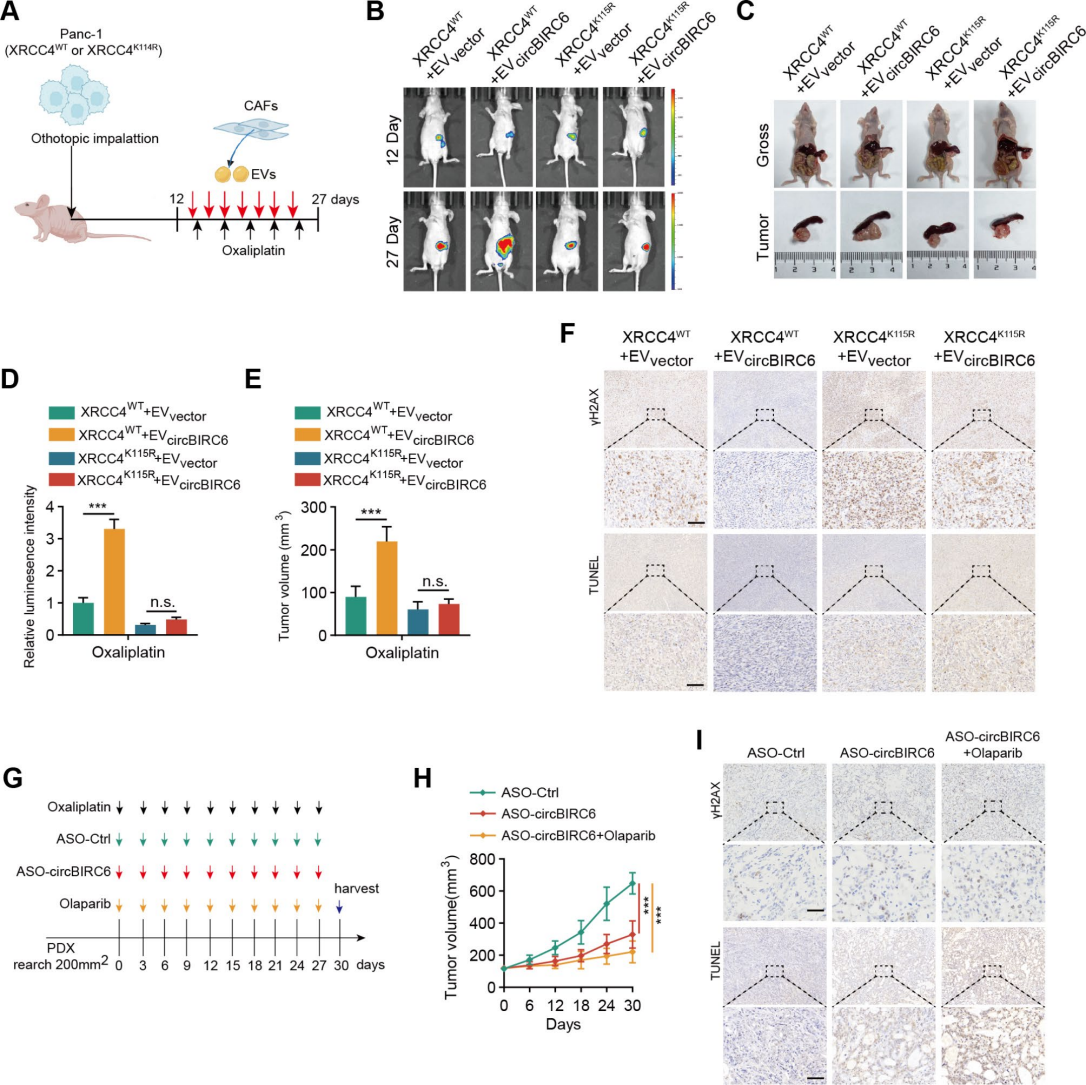
Enhanced NHEJ-Dependent DNA repair and chemoresistance in pancreatic cancer in vivo induced by CAF-derived EV-delivered circBIRC6. **(A)** Schematic representation of the orthotopic xenograft model establishment process. Panc-1 cells, transfected with either XRCC4^WT^ or XRCC4^K115R^ mutation, were injected into the pancreas of nude mice. Post-implantation (12 days), mice underwent distinct treatments at intervals indicated by colored arrows. **(B-C)** Display of representative IVIS images **(B)** and harvested pancreatic tumors **(C)** from the orthotopic xenograft model (n = 5 mice per group). **(D-E)** Quantification of the luminescence intensity **(D)** and volumetric assessment of the tumor **(E)** in the orthotopic xenograft model. **(F)** Illustrative images of immunohistochemical staining for γH2AX and TUNEL in the orthotopic xenograft model. Scale bars equate to 100 μm. **(G)** A chronological schematic for the mice treatment in the Patient-Derived Xenograft (PDX) model, with colored arrows representing various treatment time points. **(H)** Depiction of tumor growth curves (n = 5 mice per group). **(I)** Exhibiting representative images of immunohistochemical staining for γH2AX and TUNEL in the PDX model. Scale bars equate to 100 μm. Data are presented as mean ± SD. n.s., not significant. ***p < 0.001

To investigate the role of EV-packaged circBIRC6 in vivo, Panc-1 cells transduced with either XRCC4-WT or XRCC4-Mut were injected into the pancreas tail of mice, and the orthotopic xenograft mice were treated with oxaliplatin and the indicated CAF-derived EVs (Fig. 7A). All groups displayed equivalent fluorescence intensity levels at the onset of treatment, suggesting that the XRCC4 mutation did not affect tumor growth (Fig. 7B). Intravenous injection of CAF-derived EV-packaged circBIRC6 led to a reduced chemotherapy response, with larger tumor sizes (Fig. 7B-E). Additionally, levels of DSBs and apoptosis were reduced in xenografts treated with EV-packaged circBIRC6 (Fig. 7F). Moreover, XRCC4^115R^ mutation dramatically abolished the effect of CAF-derived EV-packaged circBIRC6 (Fig. 7B-F).

To assess the potential therapeutic value of the circBIRC6/XRCC4 axis in a more patient-relevant in vivo context, we established patient-derived xenograft (PDX) models from oxaliplatin-resistant patients and treated them with antisense oligonucleotide (ASO) inhibitors targeting circBIRC6 (Fig. 7G). As expected, treatment of ASO-circBIRC6 significantly improved the chemotherapeutic response. Moreover, combining oxaliplatin with ASO-circBIRC6 and Olaparib dramatically enhanced the chemotherapeutic response (Fig. 7H). IHC analysis revealed that treatment with ASO-circBIRC6 or ASO-circBIRC6 combined with Olaparib increased the levels of DSBs and apoptosis (Fig. 7I). Collectively, these data suggest that CAF-derived EV-packaged circBIRC6 promotes oxaliplatin resistance via SUMOylated XRCC4 in vivo.

### circBIRC6 is associated with DNA damage and oxaliplatin resistance in PDAC patients

**Fig. 8:**
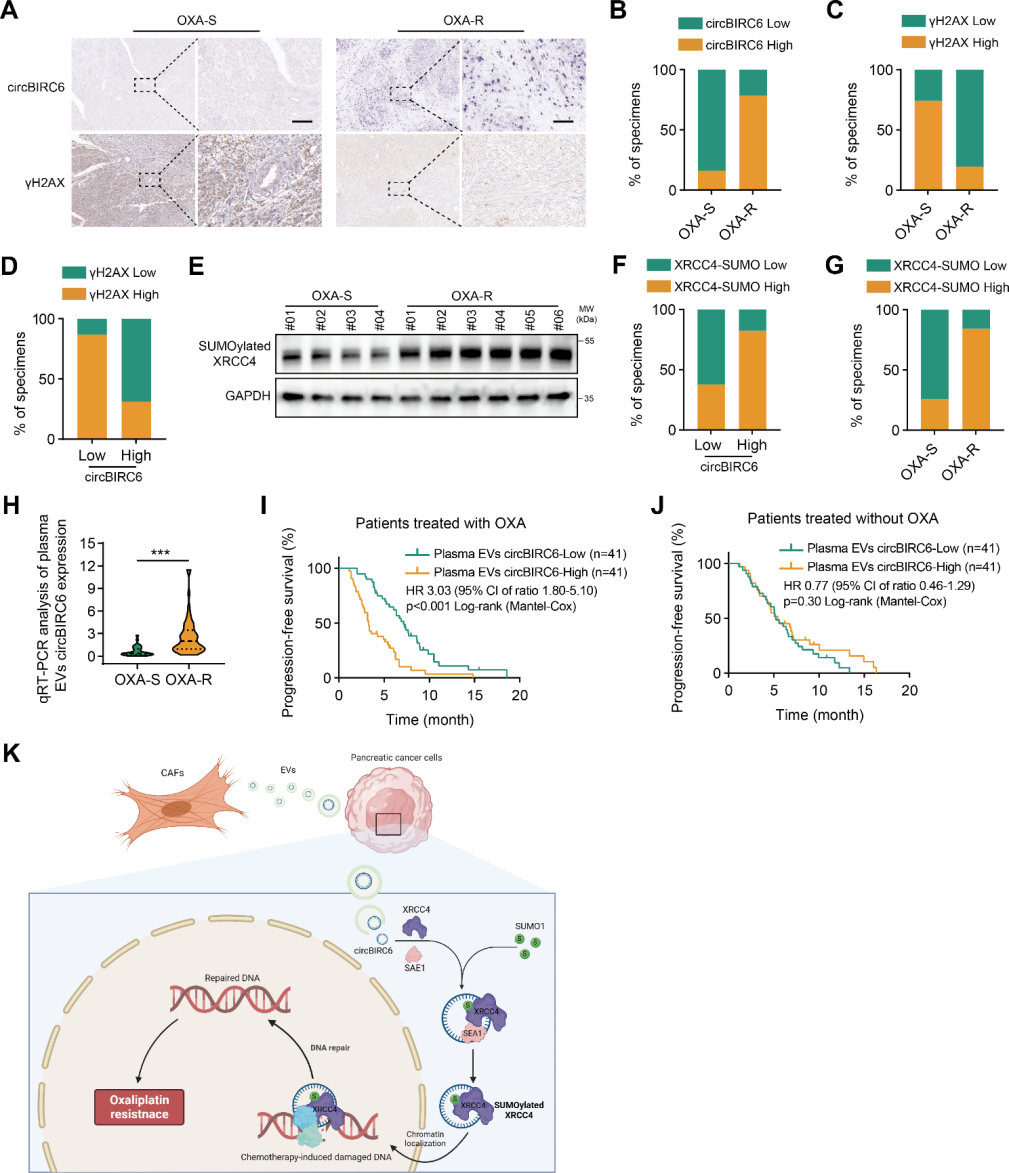
EV-packaged circBIRC6 association with DNA repair and chemoresistance in pancreatic cancer patients. **(A)** Depiction of representative ISH results for circBIRC6 and IHC for γH2AX in oxaliplatin-resistant (OXA-R, n = 51) and oxaliplatin-sensitive (OXA-S, n = 31) pancreatic cancer samples. Scale bars, 100 μm. **(B-C)** Quantitative breakdown of the specimen percentages featuring low or high expression of circBIRC6 **(B)** and γH2AX **(C)** across oxaliplatin-resistant and oxaliplatin-sensitive groups. **(D)** Quantification of the specimen percentages with low or high γH2AX expression within groups categorized by low or high circBIRC6 expression. **(E)** SUMOylated XRCC4, immunoprecipitated using the SUMO1 antibody, was analyzed by Western blot in OXA-R and OXA-S pancreatic cancer specimens. **(F)** Percentage distribution of samples with different SUMOylated XRCC4 levels grouped by circBIRC6 expression. **(G)** Distribution of samples based on SUMOylated XRCC4 expression within groups defined by oxaliplatin response. **(H)** Evaluation of plasma EV-packaged circBIRC6 expression using qRT–PCR in oxaliplatin-sensitive (OXA-S, n = 31) and oxaliplatin-resistant (OXA-R, n = 51) PDAC tissues. **(I)** Kaplan–Meier survival curves representing patients treated with platinum-based chemotherapy, categorized by high or low plasma EV-packaged circBIRC6 expression. **(J)** Kaplan–Meier survival curves representing patients undergoing chemotherapy without platinum, segregated by high or low plasma EV-packaged circBIRC6 expression. **(K)** Schematic illustration delineating the mechanism by which EV-packaged circBIRC6 contributes to CAFs’ promotion of oxaliplatin chemoresistance in pancreatic cancer. Data are presented as mean ± SD. ***p < 0.001

To evaluate the predictive value and clinical implications of circBIRC6 on oxaliplatin-chemotherapy response, we detected circBIRC6 expression in plasma EVs and primary tumor tissues, and DNA damage levels assessed by immunostaining for γH2AX in 82 cases of advanced PDAC receiving oxaliplatin-based chemotherapy. In oxaliplatin-resistant patients, we noticed an elevated presence of circBIRC6 and a diminished level of γH2AX in tumor tissues compared to patients who responded well to oxaliplatin treatment (Fig. 8A-C). circBIRC6 expression exhibited a negative correlation with γH2AX expression (Fig. 8D). We also measured the levels of SUMOylated XRCC4 and observed a correlation between elevated SUMOylated XRCC4 levels, increased circBIRC6 expression, and reduced responsiveness to chemotherapy. (Fig. 8E-G). Furthermore, plasma EV-packaged circBIRC6 levels were elevated in oxaliplatin-chemoresistant patients relative to oxaliplatin-responsive patients (Fig. 8H). Kaplan-Meier analysis revealed that high plasma EV-packaged circBIRC6 levels were associated with shorter PFS (Fig. 8I). Intriguingly, no significant difference in PFS was observed in patients receiving other chemotherapy regimens without oxaliplatin, regardless of plasma EV-packaged circBIRC6 levels (Fig. 8J). Taken together, our findings suggest that circBIRC6 may serve as a potential biomarker for oxaliplatin-based chemotherapeutic resistance in pancreatic cancer (Fig. 8K).

## Discussion

Over the past decades, advancements in chemotherapy have brought significant benefits for pancreatic cancer patients, yet the unresolved challenge of chemoresistance emergence following treatment remains formidable [19]. Owing to the marked desmoplastic reaction in pancreatic cancer, CAFs, as the dominant cell population in the tumor stroma, critically contribute to chemoresistance in this highly fibrotic solid malignancy [20]. We recently discovered that CAFs enhance oxaliplatin resistance in pancreatic cancer cells by promoting IL-8-driven transactivation of lncRNA UPK1A-AS1, which subsequently increases the efficiency of NHEJ repair of DNA damage [13]. However, the efficacy of current IL-8 antagonistic treatments is limited, and our understanding of how CAFs-derived circRNAs mediate platinum resistance in pancreatic cancer is not yet clear. Here, we discerned an elevation in circBIRC6 levels within CAF-derived EVs, playing a pivotal role in fostering platinum resistance in PDAC patients. This resistance is driven by SUMOylation-mediated XRCC4 nuclear translocation, amplifying the NHEJ repair process in DNA-damaged pancreatic cancer cells. Additionally, our clinical findings underscore the predictive potential of the circBIRC6/XRCC4 axis for assessing platinum resistance in pancreatic cancer patients. Our study marks a frontier in revealing the circRNA-mediated epigenetic interplay between CAFs and pancreatic cancer cells in oxaliplatin resistance. The circBIRC6/XRCC4 axis highlighted in our findings paves the way for a potentially novel therapeutic approach to conquer platinum resistance in pancreatic cancer.

Serving as pivotal transporters, EVs ferry bioactive molecules between diverse cells within the TME, thus substantially shaping the biological dynamics of cancer cells [14]. In our recent study, we discovered that the protein hnRNPA1, shuttled by EVs originating from KRAS^G12D^ pancreatic cancer cells, serves to promote lymphangiogenesis and lymph node (LN) metastasis [21]. Zeng and colleagues elucidated the significant role of exosomal circZNF91, specifically under hypoxic conditions, in orchestrating gemcitabine resistance in pancreatic cancer cells, highlighting the potential of exosomal circZNF91 as a therapeutic target [22]. Recently, our understanding of the role of circRNAs in orchestrating epigenetic modifications in pancreatic cancer cells has started to expand [23]. However, their precise mechanisms in transforming the TME remain largely unknown. Our earlier research unveiled that CAFs derived circCUL2 inducing inflammatory CAF phenotype and promoting PDAC progression [24], and circFARP1 plays a pivotal role in modifying the TME to induce GEM resistance in PDAC, emphasizing the profound impact of TME-derived circRNAs on tumoral chemoresistance [25]. Leveraging our prior RNA-seq analysis of CAFs and NAFs (GSE172096), we were the first to establish a positive correlation between circBIRC6 expression in CAFs and platinum resistance in PDAC patients. We showed that circBIRC6 in EVs from CAFs amplifies the oxaliplatin resistance of pancreatic cancer cells and organoids in vitro. Although the suppression of circBIRC6 doesn’t fully negate existing platinum resistance, the introduction of circBIRC6 via CAF-exosomes increases the IC50 value in platinum-resistant cells. This suggests a role for EV-packaged circBIRC6 in shaping acquired resistance, as opposed to intrinsic resistance, in pancreatic cancer. Notably, utilizing the PDX mouse model, we verified that si-circBIRC6 could effectively augment the sensitivity to oxaliplatin chemotherapy, thereby enhancing the chemotherapeutic response in vivo. Remarkably, our study uncovers a novel mode of circRNA intercellular communication mediated by EVs. The EV-packaged transmission of circBIRC6 from CAFs to PDAC cells constitutes a previously uncharacterized avenue of stromal-tumoral interaction, broadening our understanding of the TME’s impact on chemoresistance. Our results reveal an unrecognized facet of circRNA biology, expanding the functional repertoire of these noncoding transcripts. However, our study does not conclusively rule out the roles of other RNAs or metabolites in the mechanism of platinum resistance. We intend to further delve into the regulatory mechanisms of platinum resistance by other factors in future studies. Additionally, we will explore whether there exists a synergistic or causal relationship between circRNA and these other factors.

To provide the mechanistic insights into the role of circBIRC6 in platinum resistance in pancreatic cancer cells, our study has further delineated the mechanistic interplay between circBIRC6 and the NHEJ repair pathway. Within the realm of eukaryotic cells, the NHEJ pathway emerges as a pivotal mechanism for DSB repair due to its rapid response and wide-ranging versatility [26, 27]. Within the context of the complex NHEJ repair process, it has been reported that NHEJ factors possess a high affinity for ncRNAs, such as lncRNAs MILIP, LINP1 [28], and UPK1A-AS1 [13], and significantly contribute to the DNA damage response. Prior studies have showed DNA damage-induced ncRNAs can impact the repair process by facilitating the nucleation and molecular crowding of DDR proteins and repair proteins at DSB sites [29, 30]. Most notably, our research represents the first to identify that CAF-derived circRNAs enhance the NHEJ repair process in DNA-damaged pancreatic cancer cells. This discovery offers a fresh perspective and opens a new direction for research aimed at simultaneously targeting TME-derived ncRNAs and the NHEJ repair pathway.

Our circBIRC6-binding proteome and WB experiments point towards an interaction between circBIRC6 and XRCC4, an integral component of the NHEJ repair machinery. XRCC4 is crucial for NHEJ during the Direct ligation of the DNA ends, with the involvement of other proteins like Ligase IV (LIG4), XRCC4-like factor (XLF), and Ku80/Ku70 to effectively repair DSBs [27, 31]. In hepatocellular carcinoma (HCC), the novel lncRNA NIHCOLE acts as a scaffold and facilitates the formation of multimeric complexes involving Ku70/80, APLF, XRCC4, and DNA ligase IV to promoting the ligation efficiency of DNA DSBs [32]. A prior study revealed that SBF2-AS1, an upregulated lncRNA in glioblastoma multiforme cells, promotes temozolomide (TMZ) resistance by functioning as a ceRNA for miR-151a-3p, disinhibiting its target XRCC4 and enhancing DNA repair [33]. Our investigation elucidated that circBIRC6 intricately associates with XRCC4, facilitated by the generation of a double-stranded stem-loop configuration within its 500–545 nt region. A novel finding from our study is circBIRC6-mediated SUMOylation of XRCC4, a key player in the DNA repair mechanism. An emerging consensus in current research suggests that SUMOylation profoundly influences all major DNA repair pathways, damage avoidance mechanisms, and checkpoint responses [34]. A recent study by Maria et al. unveils two novel polySUMO2-binding modules on XRCC4, highlighting their paramount role in modulating NHEJ DNA repair and the potential regulatory implications for other DNA repair proteins [35]. This PTM of XRCC4 is a compelling revelation, emphasizing the complexity of XRCC4’s functional regulation [36]. SUMOylation appears to be a crucial prerequisite for the nuclear translocation of XRCC4. Intriguingly, we observed a marked enhancement in the interaction between XRCC4 and SAE1, facilitated by circBIRC6, which further amplified the SUMOylation of XRCC4. We establish a novel observation that the dominant SUMOylation site for XRCC4 is K115 and not the traditionally known K210. Importantly, this suggests that the nuclear translocation of XRCC4 in pancreatic cancer cells largely depends on K115. In contrast to our study, Vyacheslav and colleagues elucidated the critical role of SUMO modification at lysine 210 in XRCC4’s nuclear localization [37], while Mikoto’s team emphasized the primacy of lysine 271 [37], highlighting the fine-tuned, site-specific SUMOylation as a master regulator in dictating its nuclear localization and subsequently, the orchestration of DNA damage repair. Our study uncovering for the first time that circBIRC6 can precisely recognize unique lysine residues to boost XRCC4 SUMOylation. This allows for potent promotion of XRCC4 nuclear translocation and robust enhancement of DNA repair, even when tumor cells have constrained conventional SUMOylation sites, due to continuous replenishment from extracellular vesicles-derived circBIRC6 in the TME. Through these findings, we not only broaden the understanding of XRCC4’s PTM but also pave the way for unraveling the mechanistic intricacies of XRCC4 nuclear translocation. Given the vital role of XRCC4 in DNA repair and chemoresistance, understanding the significance of its SUMOylation could have profound implications in overcoming therapeutic resistance in pancreatic cancer.

In our study, the in vivo significance of EV-packaged circBIRC6 was assessed, underscoring its role in fostering oxaliplatin resistance in pancreatic cancer. Via manipulating the SUMOylation status of XRCC4, we showed that EV-encapsulated circBIRC6 could effectively modulate chemotherapeutic responsiveness in a xenograft mouse model, thereby providing a mechanistic link between CAF-derived EV cargo and the chemotherapeutic landscape within PDAC. This interaction highlights an underappreciated layer of TME influence on tumor cell DNA repair capacity and chemoresistance. In particular, the sensitivity of xenografts to chemotherapy could be significantly enhanced through circBIRC6 depletion or mutation of XRCC4, demonstrating the potential of targeting the circBIRC6/XRCC4 axis for therapeutic intervention. Further reinforcing the clinical relevance of our findings, the manipulation of circBIRC6 levels using antisense oligonucleotides (ASOs) in patient-derived xenografts (PDXs) led to a markedly improved chemotherapeutic response. A recent study underscores the crucial role and clinical potential of the DSB repair pathway in augmenting the efficacy of PARP inhibitors for treating drug-resistant ovarian and triple-negative breast cancer, achieved through the synergistic action of SIK2 inhibitors, which both impairs the DSB repair pathway and amplifies PARP inhibitor sensitivity [38]. Remarkably, our finding not only supports the viability of a translational application of our insights but also sets a path towards a synergistic therapeutic approach, using combined oxaliplatin, ASO-circBIRC6, and PARP inhibitor (Olaparib) treatment.

To extend our observations into a clinical context, we investigated the predictive value of circBIRC6 expression in patients with PDAC. We found a negative correlation between circBIRC6 and γH2AX levels, hinting at a diminished DNA damage response in tumors with elevated circBIRC6 expression. Moreover, patients exhibiting higher EV-packaged circBIRC6 levels demonstrated poorer outcomes following oxaliplatin-based chemotherapy. Recently, a study by Kristensen et al. revealed that while CDR1as was absent in in vivo colon cancer cells, it was highly expressed in tumor stromal cells [39]. This underscores the critical need to determine the specific spatial expression patterns of circRNAs within the TME. From primary CAFs and tumor cell isolations and through subsequent passages, we discerned that circBIRC6, predominantly originating from CAFs, is closely linked to the shortened PFS in pancreatic cancer patients treated with platinum-based chemotherapy. Moreover, patients resistant to oxaliplatin-based chemotherapy had higher levels of EV-packaged circBIRC6, which correlated with poorer PFS. Additionally, circBIRC6 emerged as a potential biomarker of oxaliplatin resistance in our study. Higher plasma EV-packaged circBIRC6 levels were associated with oxaliplatin resistance and poorer patient outcomes. The clinical application of circBIRC6 as a non-invasive, predictive biomarker holds promise for personalized chemotherapy regimens, facilitating better therapeutic outcomes for PDAC patients.

## Conclusions

In conclusion, our study elucidates an intriguing mechanism underlying the crosstalk between CAFs and PDAC cells that results in chemoresistance, revealing a novel function for the noncoding RNA, circBIRC6, in promoting resistance to oxaliplatin. By deciphering the complex interaction between circBIRC6, XRCC4, and the process of SUMOylation, we highlight the potential of this axis as a promising therapeutic target to enhance chemosensitivity in PDAC.

### Electronic supplementary material

Below is the link to the electronic supplementary material.

Supplementary Material 1

## Data Availability

The datasets used in current study are available from the corresponding author on reasonable request.

## List of Abbreviations

ATCC: American Type Culture Collection
ASO: Antisense oligonucleotide
CR: Complete Response
CM: Culture medium
CAFs: Cancer-associated fibroblasts
circRNAs: Circular RNAs
cDNA: Complementary DNA
Co-IP: Co-Immunoprecipitation
DSB: Double-strands DNA break
DMEM: Dulbecco’s Modified Eagle Medium
EVs: Extracellular vesicles
FM: Fibroblast medium
FBS: Fetal bovine serum
FISH: Fluorescence in situ hybridization
HCC: Hepatocellular carcinoma
IHC: Immunohistochemistry
ISH: In situ hybridization
IVIS: In vivo imaging system
KEGG: Kyoto Encyclopedia of Genes and Genomes
LIG4: Ligase IV
LN: Lymph node (LN)
NAFs: Normal adjacent tissue-associated fibroblasts
NHEJ: Non-homologous end joining
PDAC: Pancreatic ductal adenocarcinoma
PARP: Poly ADP-ribose polymerase
PDX: Patient-derived xenograft
PR: Partial Response
PD: Progressive Disease
PFS: Progression-free survival
PTMs: Posttranslational modifications
qRT‑PCR: Quantitative real‑time PCR
RBPs: RNA-binding proteins
RIP: RNA immunoprecipitation
RECIST: Response Evaluation Criteria in Solid Tumors
SD: Stable Disease
TME: Tumor microenvironment
TMZ: Temozolomide
XLF: XRCC4-like factor
