# Supplementary material for: Extracellular vesicle-packaged circBIRC6 from cancer-associated fibroblasts induce platinum resistance via SUMOylation modulation in pancreatic cancer
Source: J Exp Clin Cancer Res. 2023 Nov 28;42:324. doi: 10.1186/s13046-023-02854-3 (PMC10683239; doi:10.1186/s13046-023-02854-3)
Supplement: Supplementary file 1 — Supplementary Material 1 [file 13046_2023_2854_MOESM1_ESM.docx]

**Supplemental Figures**


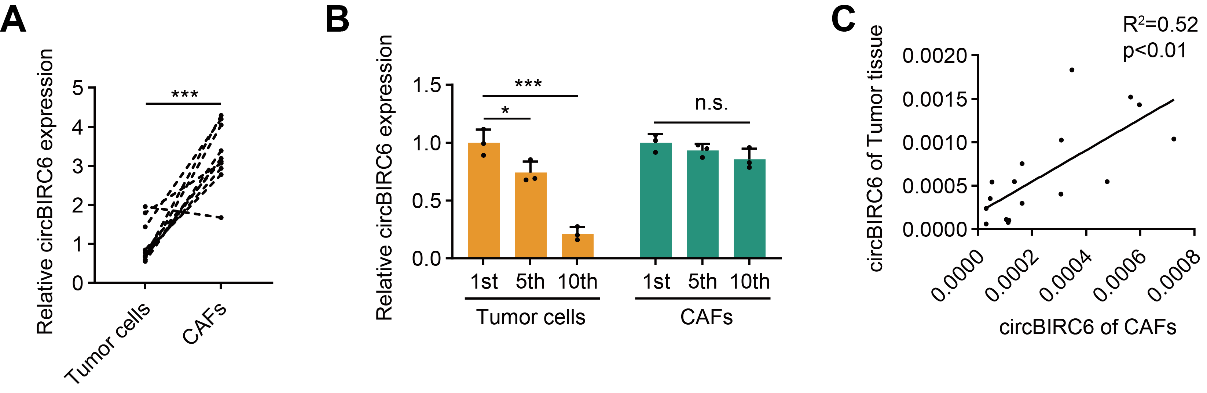


**Figure S1. Correlation of circBIRC6 with response to oxaliplatin-based chemotherapy.**

(A) qRT-PCR analysis of circBIRC6 expression in paired CAFs and tumor cells isolated from PDAC patients (n=10) received oxaliplatin-based chemotherapy.

(B) qRT-PCR analysis of circBIRC6 expression in different passages of CAFs and tumor cells.

(C) Correlation analysis of circBIRC6 expression of tumor tissue and CAFs.

Data are presented as mean ± SD. n.s., not significant. *p < 0.01, ***p < 0.001.


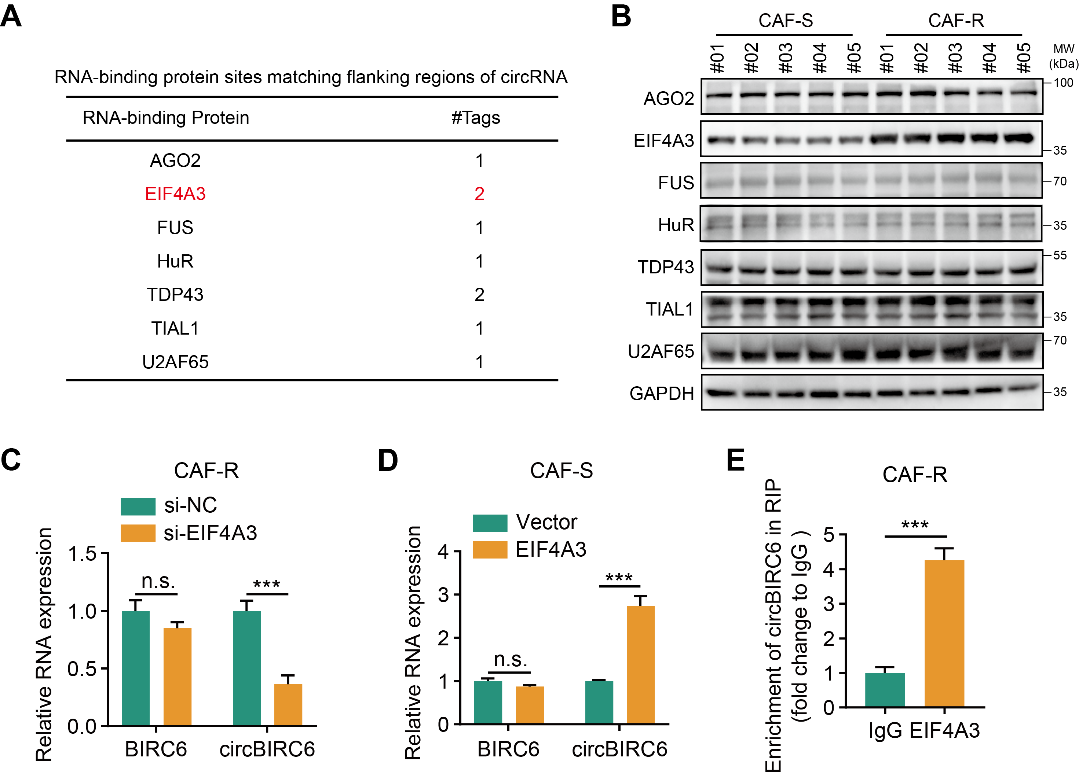


**Figure S2. EIF4A3 promotes the cyclization of circBIRC6.**

(A) Seven RNA binding proteins were predicted with the circInteractome database.

(B) Western blot analysis of Seven RNA binding proteins (AGO2, EIF4A3, FUS, HuR, TDP43, TIAL1 and U2AF65) in CAFs isolated from oxaliplatin-sensitive (CAF-S) and oxaliplatin-resistant patients (CAF-R).

(C-D) qRT-PCR analysis of circBIRC6 and BIRC6 expression in CAFs depleted (C) or overexpressing (D) of EIF4A3.

(E) RIP assay validating circBIRC6's interaction with EIF4A3 in CAF-R.

Data are presented as mean ± SD. n.s., not significant. ***p < 0.001.


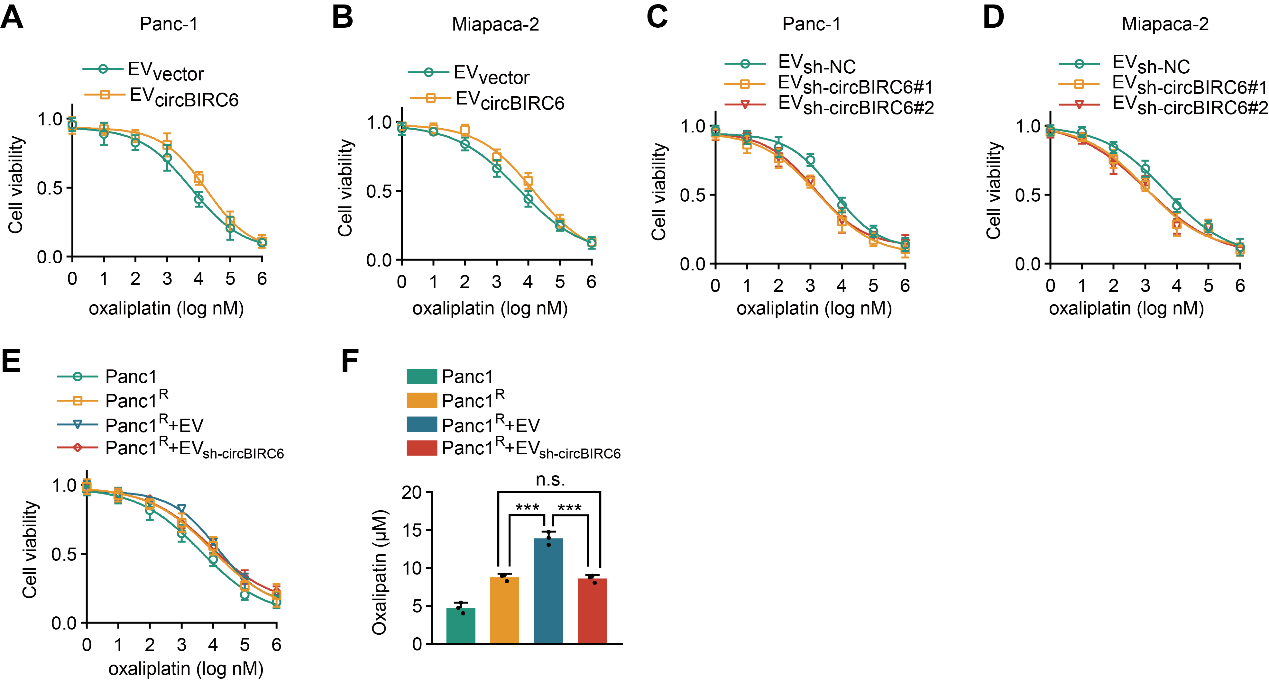


**Figure S3. circBIRC6 delivery via EVs amplifies oxaliplatin resistance in pancreatic cancer cells.**

(A-D) Panc-1 and MIAPaCa-2 cells were cultured with CAF-derived EV as indicated for a period of one week. The harvested pancreatic cancer cells were subsequently exposed to oxaliplatin for 48 hours. Oxaliplatin IC50 of Panc-1 and Panc-1R were determined by constructing a dose–response curve.

(E-F) Panc-1 and established oxaliplatin resistant Panc-1 (Panc-1^R^) were cultured with CAF-derived EV as indicated for one week. The harvested pancreatic cancer cells were subsequently exposed to oxaliplatin for 48 hours. Oxaliplatin IC50 of Panc-1 and Panc-1^R^ were determined by constructing a dose–response curve.

Data are presented as mean ± SD. n.s., not significant. ***p < 0.001.


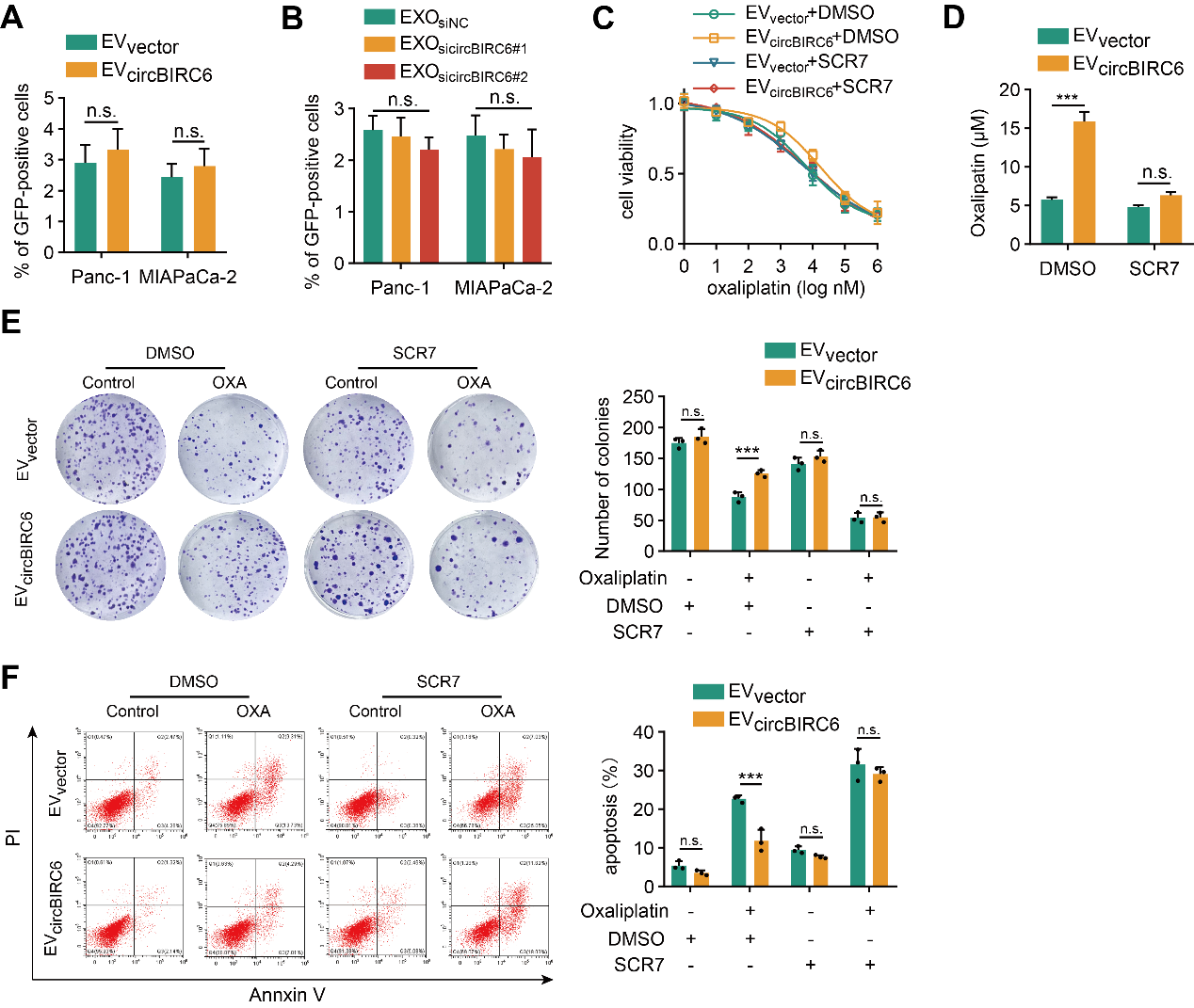


**Figure S4. EV-packaged circBIRC6 augments oxaliplatin resistance via activation of NHEJ-dependent DNA repair in pancreatic cancer.**

(A-B) The pimEJ5-GFP reporter assay was utilized to determine the NHEJ-mediated DNA repair efficiency in the collected tumor cells.

(C-F) Panc-1cells, post-treatment with EVs from circBIRC6-overexpressing CAFs and exposed to oxaliplatin, were allowed to recover with or without 10 μM SCR7. (C-D) Oxaliplatin IC50 of Panc-1 and Panc-1^R^ were determined by constructing a dose–response curve. (E) Imaging and quantification of colonies formed by the treated pancreatic cancer cells. (F) Imaging and quantification of apoptosis in treated pancreatic cancer cells through flow cytometry.

Data are presented as mean ± SD. n.s., not significant. ***p < 0.001.


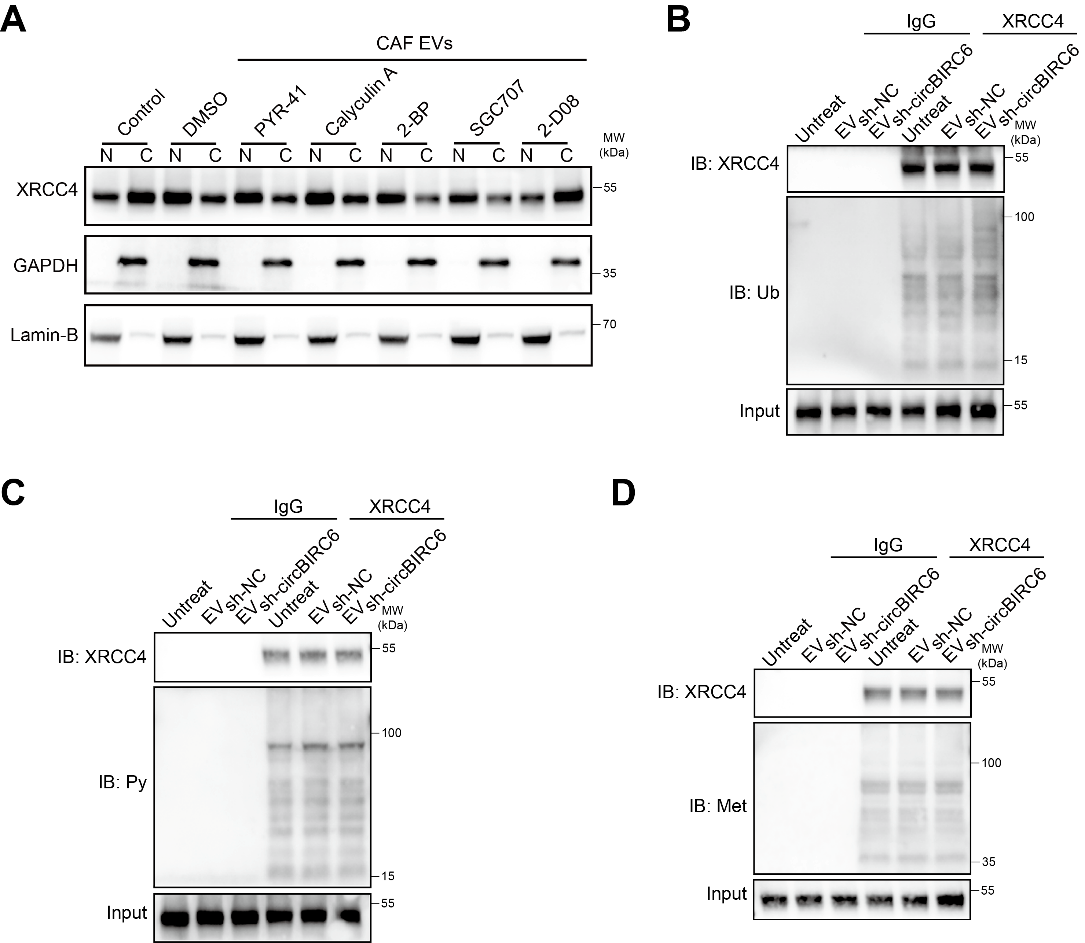


**Figure S5. circBIRC6 enhances SUMOylation of XRCC4 at the K115 residue.**

(A) Western blot was performed to evaluate the distribution of XRCC4 within the cytoplasmic and nuclear compartments of Panc-1 cells treated with CAF-isolated EVs and the specified inhibitor for Protein Translational Modifications (PTM).

(B) Co-IP analysis of ubiquitin of XRCC4 in Panc-1 cells treated with EVs from CAFs transfected with lenti-circBIRC6-shRNA or lenti-NC-shRNA.

(C) Co-IP analysis of phosphorylation of XRCC4 in Panc-1 cells treated with EVs from CAFs transfected with lenti-circBIRC6-shRNA or lenti-NC-shRNA.

(D) Co-IP analysis of Methylation of XRCC4 in Panc-1 cells treated with EVs from CAFs transfected with lenti-circBIRC6-shRNA or lenti-NC-shRNA.

**Supplemental Tables**

**Table S1. primer used in the experiment**

| Gene | Sequence(5’-3’) |
| --- | --- |
| circBIRC6-F (convergent) | TTGTTGGTAGGACTGCGGAC |
| circBIRC6-R (convergent) | TTCCAGCTCACCTTTCCGTC |
| circBIRC6-F (divergent) | GCACTGAGGAACAGGACACAT |
| circBIRC6-R (divergent) | CACCTGGTTTAGCTTTGGTGC |
| GAPDH-F (convergent) | GTCATCCCTGAGCTGAACGG |
| GAPDH-R (convergent) | GTCAAAGGTGGAGGAGTGGG |
| GAPDH-F (divergent) | CACCACACTGAATCTCCCCT |
| GAPDH-R (divergent) | ATTTCCTTCCCGGTTGCAAC |
| U6-F | CTCGCTTCGGCAGCACA |
| U6-R | AACGCTTCACGAATTTGCGT |
| XRCC4-F | GGACATCAAACAAGAAGGGGAAACT |
| XRCC4-R | AGCTGAAGCCAACCCAGAGA |

**Table S2. probes and oligonucleotides used in the experiments**

| Gene | Sequence(5’-3’) | Application |
| --- | --- | --- |
| Cy3-circBIRC6 | CACCTGTCCA CCTGGTTTAG CTTTGGTGCA TGCACACAGC | FISH |
| double-DIG-circBIRC6 | GTCCACC+TGGTTTAGCT+TTGGTGCA+TGCACA | ISH |
| sh-circBIRC6#1 | TGTGCATGCACCAAAGCTAAA | shRNA |
| sh-circBIRC6#2 | CAAAGCTAAACCAGGTGGACA | shRNA |
| si-SAE1#1-sense | AGACAACGAUGGUCAAAAATT | siRNA |
| si-SAE1#1-antisense | UUUUUGACCAUCGUUGUCUTT |  |
| si-EIF4A3-sense | GAGCAGAUUUACGAUGUAUTT | siRNA |
| si-EIF4A3-antisense | AUACAUCGUAAAUCUGCUCTT |  |

**Table S3. Antibody used in this study.**

| Antibody | Application | Source |
| --- | --- | --- |
| XRCC4 Rabbit Polyclonal antibody | WB, 1:1000  IP, 1:50 | 15817-1-AP, proteintech |
| SUMO1 Mouse Monoclonal antibody | WB, 1:1000  IP, 1:50 | 67559-1-Ig, proteintech |
| TSG101 Rabbit Monoclonal antibody | WB, 1:1000 | ab125011, abcam |
| CD9 Rabbit Monoclonal antibody | WB, 1:1000 | ab92726, abcam |
| Gamma H2A.X Rabbit Polyclonal antibody | WB, 1:5000  IF/IHC, 1:250 | ab81299, abcam |
| SAE1 Rabbit Monoclonal antibody | WB, 1:5000  IP, 1:50 | ab185552, abcam |
| Histone-H3 Rabbit Polyclonal antibody | WB, 1:2000 | 17168-1-AP, proteintech |
| Lamin B1 Rabbit Monoclonal antibody | WB, 1:1000 | 17416, CST |
| EIF4A3 Rabbit Polyclonal antibody | WB, 1:1000  IP, 1:50 | 17504-1-AP, proteintech |
| AGO2 Mouse Monoclonal antibody | WB, 1:1000 | 67934-1-Ig |
| FUS/TLS Rabbit Monoclonal antibody | WB, 1:1000 | 67840S, CST |
| HuR Mouse Monoclonal antibody | WB, 1:1000 | ab136542, abcam |
| TIAR Mouse Monoclonal antibody | WB, 1:2000 | 17504-1-AP, proteintech |
| TDP43 Rabbit Monoclonal antibody | WB, 1:2000 | 10782-2-AP, proteintech |
| U2AF65 Rabbit Monoclonal antibody | WB, 1:2000 | 15624-1-AP, proteintech |
| Phospho-Serine/Threonine Rabbit Monoclonal antibody | WB, 1:2000 | 530893, Zenbio |
| Ubiquitin Mouse Monoclonal antibody | WB, 1:1000 | 3936S, CST |
| Pan Mono-Methyl lysine Rabbit Polyclonal antibody | WB, 1:1000 | A18293, ABclonal |
| GAPDH（MC4）Mouse Monoclonal Antibody | WB, 1:5000 | RM2002, Beijing Ray  Antibody Biotech |
| Goat anti-Mouse IgG(H+L)-HRP | WB, 1:5000 | RM3001, Beijing Ray Antibody Biotech |
| Goat anti-Rabbit IgG(H+L)-HRP | WB, 1:5000 | RM3002, Beijing Ray Antibody Biotech |
| Goat anti-rabbit Alexa Fluor 488 | IF, 1:250 | ab150077, abcam |
